# Transient receptor potential ion channel TRPM2 promotes AML proliferation and survival through modulation of mitochondrial function, ROS, and autophagy

**DOI:** 10.1038/s41419-020-2454-8

**Published:** 2020-04-20

**Authors:** Shu-jen Chen, Lei Bao, Kerry Keefer, Santhanam Shanmughapriya, Longgui Chen, John Lee, JuFang Wang, Xue-Qian Zhang, Iwona Hirschler-Laszkiewicz, Salim Merali, Carmen Merali, Yuka Imamura, Sinisa Dovat, Muniswamy Madesh, Joseph Y. Cheung, Hong-Gang Wang, Barbara A. Miller

**Affiliations:** 1 Department of Pediatrics, The Pennsylvania State University College of Medicine, P.O. Box 850, Hershey, PA 17033 USA; 2 Department of Molecular Genetics and Medical Biochemistry, Temple University, Philadelphia, PA 19140 USA; 3 The Center of Translational Medicine, Temple University School of Medicine, Philadelphia, PA USA; 4 School of Pharmacy, Temple University, Philadelphia, PA 19140 USA; 5 Department of Biochemistry and Molecular Biology, The Pennsylvania State University College of Medicine, P.O. Box 850, Hershey, PA 17033 USA; 6 Department of Pharmacology, The Pennsylvania State University College of Medicine, P.O. Box 850, Hershey, PA 17033 USA; 7 The Institute for Personalized Medicine, The Pennsylvania State University College of Medicine, P.O. Box 850, Hershey, PA 17033 USA; 8 Department of Medicine, Temple University, Philadelphia, PA 19140 USA

**Keywords:** Autophagy, Calcium signalling, Stress signalling, Acute myeloid leukaemia

## Abstract

Transient receptor potential melastatin 2 (TRPM2) ion channel has an essential function in maintaining cell survival following oxidant injury. Here, we show that TRPM2 is highly expressed in acute myeloid leukemia (AML). The role of TRPM2 in AML was studied following depletion with CRISPR/Cas9 technology in U937 cells. In in vitro experiments and in xenografts, depletion of TRPM2 in AML inhibited leukemia proliferation, and doxorubicin sensitivity was increased. Mitochondrial function including oxygen consumption rate and ATP production was reduced, impairing cellular bioenergetics. Mitochondrial membrane potential and mitochondrial calcium uptake were significantly decreased in depleted cells. Mitochondrial reactive oxygen species (ROS) were significantly increased, and Nrf2 was decreased, reducing the antioxidant response. In TRPM2-depleted cells, ULK1, Atg7, and Atg5 protein levels were decreased, leading to autophagy inhibition. Consistently, ATF4 and CREB, two master transcription factors for autophagosome biogenesis, were reduced in TRPM2-depleted cells. In addition, Atg13 and FIP200, which are known to stabilize ULK1 protein, were decreased. Reconstitution with TRPM2 fully restored proliferation, viability, and autophagy; ATF4 and CREB fully restored proliferation and viability but only partially restored autophagy. TRPM2 expression reduced the elevated ROS found in depleted cells. These data show that TRPM2 has an important role in AML proliferation and survival through regulation of key transcription factors and target genes involved in mitochondrial function, bioenergetics, the antioxidant response, and autophagy. Targeting TRPM2 may represent a novel therapeutic approach to inhibit myeloid leukemia growth and enhance susceptibility to chemotherapeutic agents through multiple pathways.

## Introduction

Increased reactive oxygen species (ROS) are found in acute myeloid leukemia (AML)^1,2^. Mitochondria are a major source of ROS, which injure tissues through protein oxidation, lipid peroxidation, and DNA oxidation and mutagenesis^3^. In malignant cells, a moderate rise in ROS may promote proliferation and metastasis by aberrantly affecting proliferative or survival pathways, whereas an excessive increase results in cell death^4^. Malignant cells produce more ROS than normal cells, and a number of chemotherapy agents including doxorubicin mediate cell death by increasing ROS above a cytotoxic threshold^5–7^. In myeloid leukemia, use of pro-oxidants or inhibition of intracellular antioxidants to increase ROS above the cytotoxic threshold has been proposed as a novel approach to optimize anti-cancer drugs^4,8,9^. Myeloid leukemia stem cell have increased sensitivity to ROS, which could be utilized in their eradication^10^.

TRP channels are members of a superfamily of cation-permeable ion channels involved in fundamental cell functions^11^. Melastatin subfamily (TRPM) members have important roles in cell proliferation and survival^12^. TRPM2, the second member of this subfamily to be cloned, is expressed in many cell types, including hematopoietic cells and mediates cation influx^3,13^. Oxidative stress (H_2_O_2_) and TNFα are extracellular signals which regulate TRPM2 through production of ADP-ribose (ADPR), which binds to the TRPM2 C-terminal NUDT9-H domain, activating the channel^3,14–17^. TRPM2 is also positively regulated by the intracellular Ca^2+^ concentration^18,19^.

The ion channel TRPM2 is highly expressed in a number of cancers^20–22^. While early studies supported the concept that TRPM2 activation induced cell death by sustained increase in intracellular calcium^17,23^ or enhanced cytokine production^24^, recent investigations concluded that physiological Ca^2+^ entry via TRPM2 channels is protective rather than deleterious, consistent with high expression in cancer^22,25–27^. TRPM2 channels protect hearts of mice from ischemia/reperfusion (I/R) injury^28,29^. A TRPM2 mutant (P1018L) was found in Guamanian amyotrophic lateral sclerosis and Parkinsonism dementia patients^30^. Unlike wild-type TRPM2 which does not inactivate, the P1018L mutant inactivates after channel opening, limiting Ca^2+^ entry and suggesting TRPM2 is necessary for normal neuronal function. TRPM2 inhibition reduced neuroblastoma growth and enhanced chemotherapy responsiveness through decreased mitochondrial function and increased ROS^21,31^.

Autophagy is required for maintenance of murine hematopoietic stem cells, and reduction of ULK1 activity, a critical kinase, decreased hematopoietic stem cell survival^32^. Impaired autophagy may initially support preleukemia development and overt leukemic transformation through stabilization of oncoproteins^32^, but once leukemia is established, autophagy promotes tumor growth, cell survival, and chemotherapy resistance^33,34^. Inhibition of autophagy is an effective approach to improve chemotherapeutic response in myeloid leukemia^32,33,35–37^. In neuroblastoma^21,31^ and gastric cancer^38^, inhibition of TRPM2 reduced autophagy, although mechanisms were not completely defined.

The role of TRPM2 in AML proliferation and chemotherapy sensitivity was examined here using myeloid leukemia cells in which TRPM2 was depleted. Major findings are as follows: (1) TRPM2 is highly expressed in AML and depletion of TRPM2 inhibits leukemia proliferation and survival in vitro and in xenografts; (2) mitochondrial function and bioenergetics are reduced and mitochondrial ROS levels elevated in TRPM2-depleted leukemia cells; (3) multiple transcription factors including CREB, ATF4, and Nrf2 are reduced in TRPM2 depletion, which contributes to increased ROS; and (4) autophagy is impaired through modulation of transcription factors CREB and ATF4, which are master transcription factors for autophagosome biogenesis, resulting in decreased ULK1, Atg7, and Atg5 and autophagocytic flux. These findings demonstrate that inhibition of TRPM2 reduces leukemia growth and increases cytotoxicity in myeloid leukemia through mitochondrial dysfunction, increased mitochondrial ROS production, reduced antioxidant response and bioenergetics, and significantly impaired autophagy.

## Results

### TRPM2 is highly expressed in AML

**Fig. 1:**
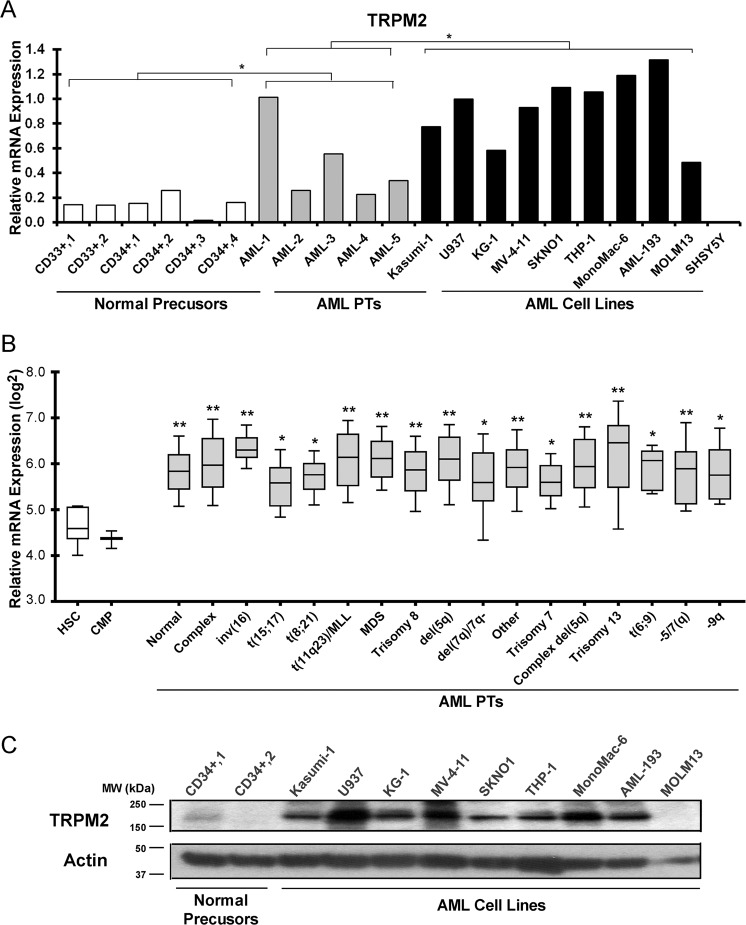
Quantitation of endogenous TRPM2 in primary AML cells, AML cell lines, CD33+ and CD34+ precursors, and normal hematopoietic progenitors. **a** RT-PCR was performed with primers specific for TRPM2 using RNA from CD33+ (*n* = 2) and CD34+ (*n* = 4) hematopoietic precursors, primary AML patient samples (*n* = 5), and human leukemia cell lines (*n* = 9). Data were normalized to expression in U937 cells. Duplicate measurements of each sample were made. Statistical differences between groups were analyzed with unpaired, two-tailed *t*-test. *p < 0.05. **b** TRPM2 expression in AML patients was examined and compared with expression in normal human bone marrow hematopoietic progenitors using the Bloodspot Database (Fig. 1b). Data were normalized as described previously^59^. Expression of TRPM2 in AML samples (*n* = 1889) from patients with no karyotypic abnormalities (normal), or different cytogenetic subgroups including aberrant complex karyotypes, inv(16), t(15;17), t(8;21), and t(11q23)/MLL was significantly greater than that found in hematopoietic stem cells (HSC, *n* = 6) or common myeloid progenitor cells (CMP, *n* = 3) isolated from normal bone marrow. The median 25–75 percentiles are boxed and the 10–90 percentiles for each group shown with bars. The median is shown with a line. Significance was assessed by one-way ANOVA. *p ≤ 0.05; **p ≤ 0.001 for AML samples in comparison to HSC and CMP. **c** Western blotting was performed with lysates from two CD34+ preparations isolated from normal human bone marrow and from nine AML cell lines in which TRPM2 mRNA was quantitated in (**a**). Blots were probed with anti-TRPM2-C or anti-actin antibodies.

High levels of TRPM2 mRNA were found in human AML cell lines compared with normal CD33+ or CD34+ precursors isolated from human bone marrow (Fig. 1a). TRPM2 mRNA measured in bone marrow samples from untreated AML patients was significantly greater than that in normal human CD33+ or CD34+ samples but less than in AML cell lines. TRPM2 mRNA quantitated in hematopoietic cells was significantly greater than that in neuroblastoma SH-SY5Y cells.

To confirm high TRPM2 expression in AML, TRPM2 expression in AML patients (*n* = 1889) and normal human bone marrow hematopoietic progenitor cells was examined using the Bloodspot data set (Fig. 1b). Expression of TRPM2 in AML samples from patients with normal karyotypes and all major AML mutational subgroups including aberrant complex karyotypes was significantly greater than that measured in normal hematopoietic stem cells (HSC) or common myeloid progenitors (CMP) (Fig. 1b). This demonstrated that TRPM2 expression was higher in AML than in normal hematopoietic cells at similar stages of differentiation.

TRPM2 protein expression was examined with western blotting of CD34+ cells and AML cell lines (Fig. 1c). TRPM2 protein expression was increased in eight of nine myeloid cell lines compared with normal precursors, similar to mRNA measured with RT-PCR.

### Generation and characterization of TRPM2-depleted U937 cells

**Fig. 2:**
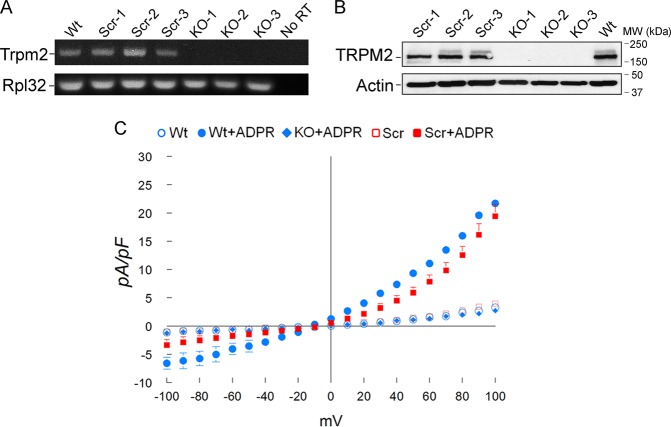
Characterization of ADPR-activated cationic currents in leukemia cells in which TRPM2 is depleted. **a** RT-PCR of TRPM2 in wild type (Wt), scrambled control (three clones, Scr-1-3), or TRPM2-depleted (KO-1-3) U937 cells. Primers to Rpl32 (ribosomal protein 32) were used as control. **b** Western blotting of lysates from three scrambled and three KO clones compared with parental wild type U937 cells. Actin was used as the loading control to confirm equivalent protein loaded/lane. **c** Whole cell patch-clamp of Wt, TRPM2-depleted, or scrambled control U937 cells. Intracellular application of ADPR (300 µM) elicited large cation currents in Wt cells (solid blue circle, *n* = 4) and scrambled (solid red square, *n* = 4), but not KO (solid blue diamond, *n* = 4) cells. Mean ± s.e.m. for each data point is shown. Error bars are not shown if they fell within the boundaries of the symbol. *p* < 0.001, two-way ANOVA for TRPM2 KO vs scrambled and wild-type control cells with ADPR. Omission of ADPR in pipette solutions resulted in background currents in scrambled (open red square, *n* = 5), and Wt control cells (open blue circle, *n* = 5), similar to KO cells with ADPR.

To examine the function of TRPM2 in myeloid leukemia, TRPM2 was depleted from U937 leukemia cells with CRISPR/Cas9 technology. TRPM2 genomic DNA sequence encoding the first 40 amino acids was deleted and the remaining sequence was frameshifted^31^. Single cell clones from TRPM2 knockout and control cells were generated and expanded. Deletion of TRPM2 in these clones was confirmed by RT-PCR (Fig. 2a) and western blotting (Fig. 2b). Three TRPM2-depleted and three scrambled control clones were randomly selected for functional experiments. Parental U937 cells (Wt), U937 cells in which TRPM2 was depleted (KO), or control cells targeted with scrambled gRNA (Scr) were subjected to patch-clamp. Intracellular application of ADPR (300 µM) elicited large inward and outward cation currents in Wt and scrambled cells, but not KO cells (Fig. 2c). ADPR-activated currents displayed the characteristic TRPM2 linear I–V relationship with reversal potential close to 0 mV^30,39^. Omission of ADPR in pipette solutions resulted in much smaller currents in Wt or Scr cells similar to those measured in KO cells in which ADPR was included in the pipette. These characteristics indicate that: (1) the ADPR-activated current in U937 cells is mediated by TRPM2; and (2) TRPM2 channel activity is absent in KO cells.

### Cell proliferation is reduced and doxorubicin sensitivity increased in TRPM2-depleted myeloid cells

**Fig. 3:**
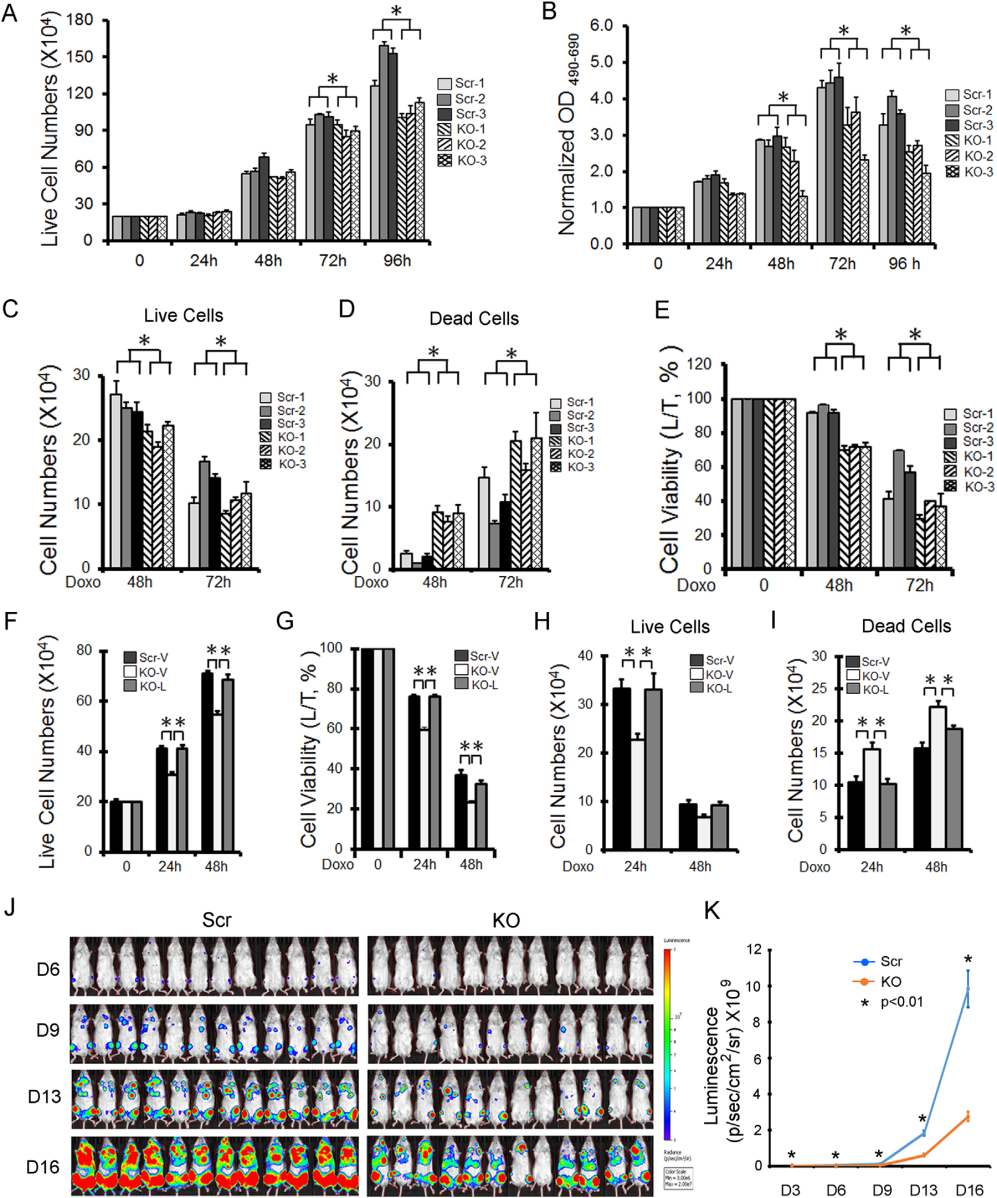
TRPM2 depletion reduces proliferation and increases doxorubicin sensitivity. **a**–**e** U937 scrambled control cells (Scr clones 1-3) or TRPM2-depleted U937 cells (KO clones 1-3) were studied at 0 to 96 h after plating (**a**, **b**). Equal numbers of cells/group were also treated with 0.1 μM doxorubicin for 48 or 72 h (**c**, **d**, **e**). Cell proliferation was measured by trypan blue exclusion (**a**) or XTT assay (**b**), and viability after exposure to doxorubicin by trypan blue (**c**, **d**, **e**). Results are expressed as live (**a**, **c**) or dead cell number (**d**), percent viability (**e**), or normalized OD reading of plated cells (**b**). Values are means ± s.e.m. for one experiment of two (**a**, **c**–**e**, *n* = 18) with trypan blue exclusion and means ± s.e.m. from three combined experiments performed with XTT (**b**, *n* = 24). Data for the second experiment with trypan blue is shown in Supplemental Information Fig. 1(a, c–e). (**a**–**e)**, *p ≤ 0.0001; group effect, Scr vs KO, two-way ANOVA. **f–i** TRPM2 reconstitution. TRPM2-depleted U937 cells were stably transfected with empty vector (KO-V) or wild type TRPM2 (KO-L). Proliferation over 0-48 h (**f**), and viability (**g**) and live vs dead cell number (**h**, **i**) 24 and 48 h after treatment with 0.3 µM doxorubicin were measured. One experiment of two quantitated with trypan blue exclusion using pooled clones for TRPM2 reconstitution is shown here (mean ± s.e.m., *n* = 8). (**f**–**i)**, *p < 0.008, subgroup analysis, Bonferroni correction, two-way ANOVA. The second experiment is shown in Supplemental Information Fig. 1f–i. (**j**, **k**) NSG mice were injected intravenously with 8 × 10^3^ U937 cells depleted of TRPM2 (KO) or scrambled control cells transfected with the pCDH-EF1-Luc2-P2A-tdTomato vector. Mice were injected with luciferin and luminescence quantitated every 2–3 days with the IVIS System for 16 days. Mice injected with cells depleted of TRPM2 showed significantly reduced leukemia growth compared with scrambled controls (*n* = 12 mice/group/experiment). Three experiments were performed. Mean ± s.e.m. for each time point from one experiment is shown. *p ≤ 0.01, unpaired, two-tailed *t*-test. Two additional experiments are shown in Supplemental Information Fig. 2.

Cell proliferation quantified by trypan blue exclusion (Fig. 3a) or XTT analysis (Fig. 3b) was significantly reduced in TRPM2-depleted U937 cells compared with scrambled control clones. Comparing Scr and KO cells, there were significant group (*p* < 0.0001), time (*p* < 0.0001), and group × time interaction (*p* < 0.0001) effects, demonstrating that differences in proliferation were amplified with time. Doxorubicin treatment resulted in significantly reduced number of live cells (Fig. 3c, *p* < 0.0001) and increased number of dead cells (Fig. 3d, *p* < 0.0001) at 48 or 72 h in TRPM2-depleted cells compared with scrambled controls, measured with trypan blue exclusion. Cell viability was significantly lower in KO cells after treatment (Fig. 3e, *p* < 0.0001).

To eliminate the possibility of off-target effects during CRISPR/Cas9 treatment, TRPM2-depleted U937 cells were transfected with empty vector or wild type TRPM2. Expression of TRPM2 but not empty vector restored cell proliferation (Fig. 3f). Cell viability, and number of live and dead cells after doxorubicin in reconstituted TRPM2-depleted cells was restored to that in scrambled controls (Fig. 3g–i). This demonstrates the absence of significant off-target effects and the critical role of TRPM2 in AML proliferation and doxorubicin sensitivity.

To examine the importance of TRPM2 in leukemia growth in vivo, NSG female mice were injected intravenously with 8 × 10^3^ TRPM2-depleted, or scrambled control U937 cells, infected with the pCDH-EF1-Luc2-P2A-tdTomato vector. These cells showed stable and equivalent luciferase expression prior to injection. Leukemic growth was measured for 16-17 days. Mice injected with TRPM2-depleted cells demonstrated significantly reduced leukemia compared with scrambled controls in three experiments (Fig. 3j, k; Supplemental Information Fig. 2).

### Mitochondrial function is significantly reduced in TRPM2-depleted AML cells

**Fig. 4:**
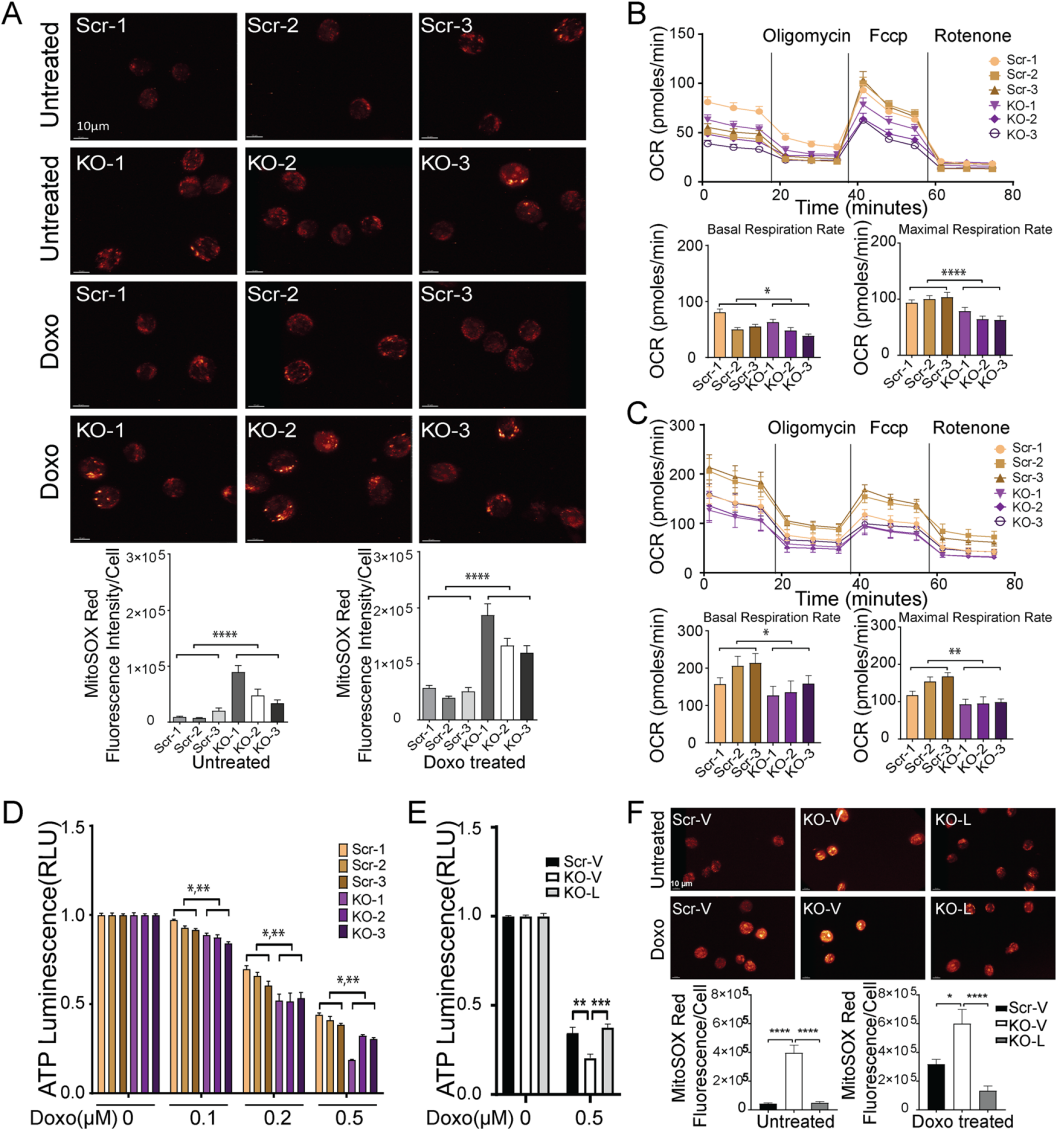
Mitochondrial function is reduced in TRPM2 depletion. **a** Mitochondrial ROS were quantitated in TRPM2-depleted (KO-1-3) and scrambled control U937 cells (Scr-1-3) with MitoSOX Red and confocal microscopy at baseline and 24 h after treatment with 0.1 μM doxorubicin. Fluorescence intensity was quantitated in a minimum of 2–10 cells/field in at least 10 fields/group in each experiment. A sample field is shown for untreated (top) and doxorubicin treated cells (bottom) from each group. Mean ± s.e.m. fluorescence intensity for each clone of untreated cells calculated from three experiments is shown below on the left. The fluorescence intensity (mean ± s.e.m.) for each clone of doxorubicin treated cells from five experiments is shown below on the right. ****p < 0.0001, paired, two-tailed *t*-test. **b** O_2_ consumption rate was measured in U937 cells depleted of TRPM2 (KO-1-3) and scrambled control cells (Scr-1-3). TOP: After basal OCR was obtained, oligomycin (2 µM) was added to inhibit F_0_F_1_ATPase (Complex V). The uncoupler FCCP (0.125 µM) was then added and maximal OCR was measured. Each point in the traces represents the average of 6 different wells. Basal (bottom left) and maximal (bottom right) mean ± s.e.m. OCRs of the 6 groups of cells calculated from four experiments are shown (*n* = 24/group). *p < 0.02 basal, ****p < 0.0001 maximal, paired, two-tailed *t*-test. **c** O_2_ consumption rate was measured in U937 cells depleted of TRPM2 (KO-1-3) and scrambled control cells (Scr-1-3) 24 hours after treatment with 0.1 μM doxorubicin. Mean ± s.e.m. OCRs of the 6 groups of cells from three experiments were calculated (*n* = 18/group). *p ≤ 0.02 basal, **p < 0.002 maximal, paired, two-tailed *t*-test. **d** ATP levels were measured in TRPM2-depleted (KO-1-3) or scrambled control cells (Scr-1-3) with the Cell Titer Glow Assay 24 h after treatment with 0.1–0.5 μM doxorubicin. Results are expressed as ATP luminescence units. Means ± s.e.m. were calculated from 2 experiments (*n* = 7). *p < 0.0001 group effect; **p < 0001 group × doxorubicin exposure time interaction effect, two-way ANOVA. **e** ATP levels were measured in TRPM2-depleted cells (KO-1) transfected with empty vector (KO-V) or wild type full length TRPM2 (KO-L). Control was scrambled cells transfected with vector (Scr-V). Means ± s.e.m. were calculated from two experiments (*n* = 6). **p < 0.002 Scr-V vs KO-V; ***p < 0.0002, KO-V vs KO-L, two-way ANOVA. **f** ROS levels in scrambled control cells expressing empty vector (Scr-V) or TRPM2-depleted cells expressing empty vector (V) or TRPM2 (L) were measured with Mitosox Red. Fluorescence intensity was quantitated in a minimum of 1–13 cells/field for untreated cells (*n* = 8–11 fields/group) and in 15 fields/group for doxorubicin treated cells. Mean ± s.e.m. fluorescence intensity for each clone is shown. Differences were analyzed with one-way ANOVA (*p < 0.05; ****p < 0.0001).

Because ROS were increased in neuroblastoma cells depleted of TRPM2^31^, mitochondrial ROS were quantitated in TRPM2-depleted myeloid leukemia cells using MitoSOX Red and confocal microscopy. Untreated TRPM2-depleted U937 cells showed significantly greater mitochondrial ROS than scrambled control cells. ROS dramatically increased in TRPM2-depleted cells following doxorubicin treatment (Fig. 4a).

To further examine mitochondrial function in TRPM2-depleted myeloid leukemia cells, oxygen consumption rates (OCRs) and ATP production were quantitated. Basal and maximal OCR were significantly lower in TRPM2-depleted cells compared with scrambled control cells (Fig. 4b). Similarly, following doxorubicin treatment, basal and maximal OCRs were significantly lower in TRPM2-depleted cells compared with controls (Fig. 4c). ATP levels were significantly lower in TRPM2-depleted cells compared with scrambled controls after treatment with doxorubicin (Fig. 4d), and were fully restored after TRPM2 reconstitution (Fig. 4e). In addition, ROS levels were significantly reduced to the level in controls (Fig. 4f). This demonstrates the critical role of TRPM2 in bioenergetics and in regulation of mitochondrial ROS in AML.

**Fig. 5:**
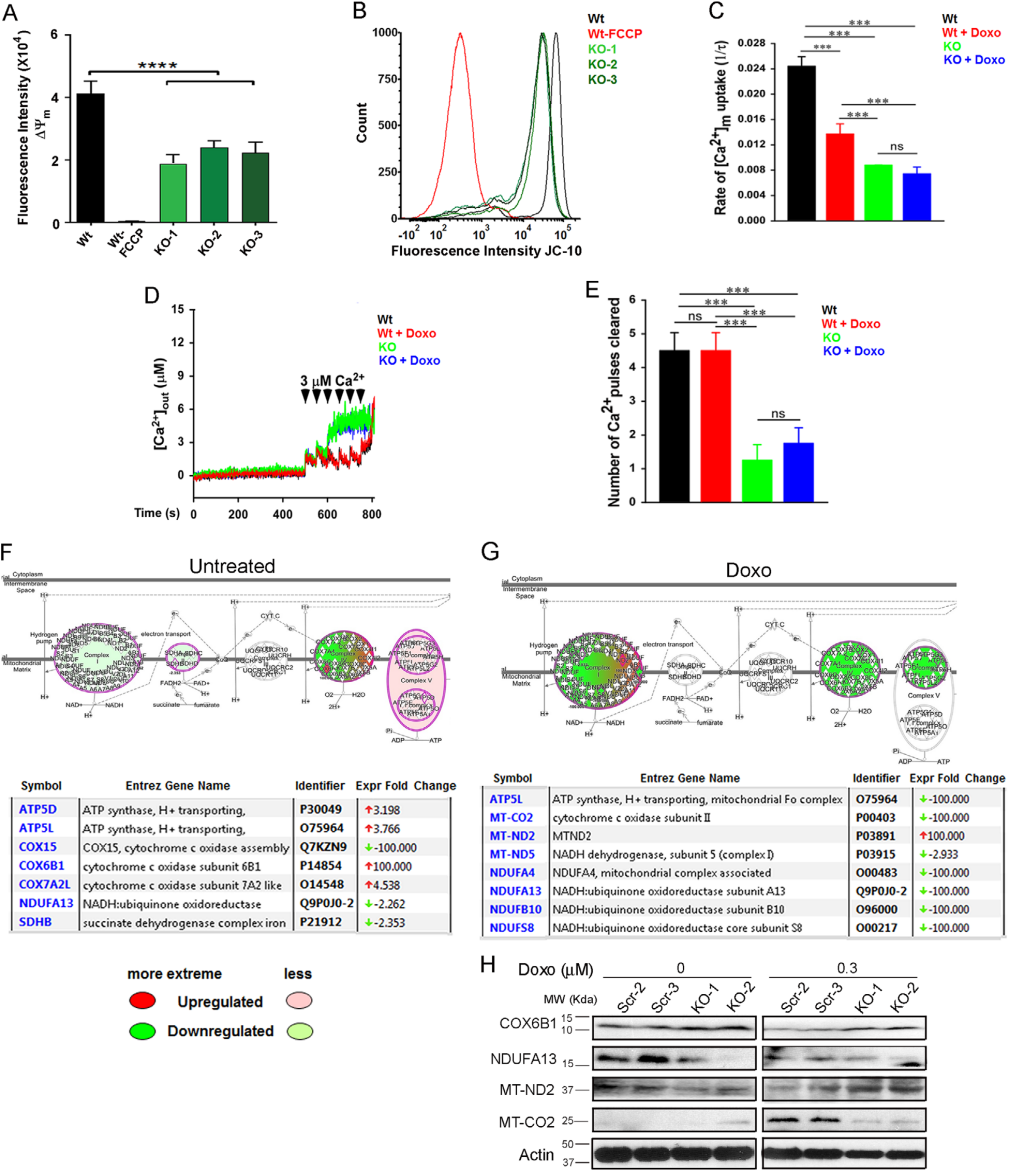
TRPM2 KO alters mitochondrial membrane potential (Δψm) and mitochondrial calcium uptake. **a** Quantification of Δψ_m_ with JC-10 in Wt and three KO clones, and Wt cells pretreated with FCCP as a control. Mean ± s.e.m. was calculated for each group from three experiments (*n* = 5/group). ****p ≤ 0.0001, paired, two-tailed *t*-test. **b** Individual traces of Δψ_m_ in Wt and KO cells. **c** Quantification of the rate of [Ca^2+^]_m_ uptake in Wt and KO cells with or without doxorubicin as a function of decrease in bath Ca^2+^ over 250 s after a single extramitochondrial Ca^2+^ pulse (3 µM). Data represent mean ± s.e.m.; ***p < 0.001; *n* = 7/group. **d** Traces of [Ca^2+^]_out_ (extramitochondrial) in Wt and KO cells with or without doxorubicin during six extramitochondrial Ca^2+^ pulses. **e** Quantification of [Ca^2+^]_m_ uptake as a function of decrease in bath Ca^2+^ calculated as number of Ca^2+^ pulses cleared after six extramitochondrial Ca^2+^ pulses (3 µM). Data represent mean ± s.e.m.; ***p < 0.001; *n* = 7. **f**, **g** Ingenuity Pathways Analysis of changes in mitochondrial proteins in TRPM2-depleted leukemia cells. Global label-free proteomics analysis was performed using U937 cells in which TRPM2 was depleted with CRISPR, or scrambled control cells. Cell were untreated (**f**) or treated with doxorubicin (**g**). Results demonstrated a decrease in Complex I, II, and IV proteins and an increase in Complex V in untreated KO cells. Color code for intensity and direction of differences in KO is shown below **f**. After doxorubicin, there was a further decline in Complex I and IV proteins, and a decline in Complex V proteins in the KO. Individual electron transport proteins which showed the greatest modulation by TRPM2 are identified in boxes (**f**, **g**). **h** Changes in COX6B1, NDUFA13, MT-ND2, and MT-CO2 were confirmed by western blotting of two scrambled (2,3) and two KO clones (1,2) (see Supplemental Information Fig. 3).

Mitochondrial function was further evaluated by measurement of mitochondrial membrane potential (Δψ_m_) and mitochondrial calcium uptake. Δψ_m_ was lower in KO cells compared with parental control cells measured with JC-10 (Fig. 5a, b). Results were confirmed with TMRE (tetramethylrhodamine, ethyl ester; data not shown). The rate of mitochondrial Ca^2+^ uptake after a single extramitochondrial 3 µM Ca^2+^ pulse was significantly lower in TRPM2 KO cells compared with wild type, both before and after doxorubicin treatment (Fig. 5c). After exposure to six extramitochondrial Ca^2+^ pulses, wild type mitochondria took up significantly more Ca^2+^ than TRPM2-depleted, as indicated by clearance of more cytoplasmic Ca^2+^ pulses (Fig. 5d, e).

To determine whether TRPM2 depletion may affect mitochondrial function and ROS levels through modulation of mitochondrial electron complex proteins, mitochondrial protein expression was assessed with label-free proteomics analysis. This demonstrated reduction in proteins involved in mitochondrial electron transport Complexes I, II, and IV in TRPM2-depleted U937 cells compared with scrambled controls (Fig. 5f). After doxorubicin treatment, the differential expression became greater in Complexes I and IV, and also involved proteins in Complex V (Fig. 5g). Proteins with greatest changes are highlighted in Fig. 5f, g. Changes in mitochondrial proteins were confirmed including increase in cytochrome c oxidase subunit 6B1 (COX6B1) and decrease in NADH:ubiquinone oxidoreductase (NDUFA13) in KO cells, and after doxorubicin an increase in mitochondrial NADH dehydrogenase subunit (MT-ND2) and decrease in cytochrome c oxidase subunit II (MT-CO2) in the KO (Fig. 5h). Together, results demonstrate significant impairment of mitochondrial function, reduced cellular bioenergetics, and increased ROS, contributing to reduced cell proliferation and survival, and increased doxorubicin sensitivity.

### Transcription factors HIF-1/2α, Nrf2, CREB, and ATF4 are reduced in TRPM2-depleted myeloid cells

**Fig. 6:**
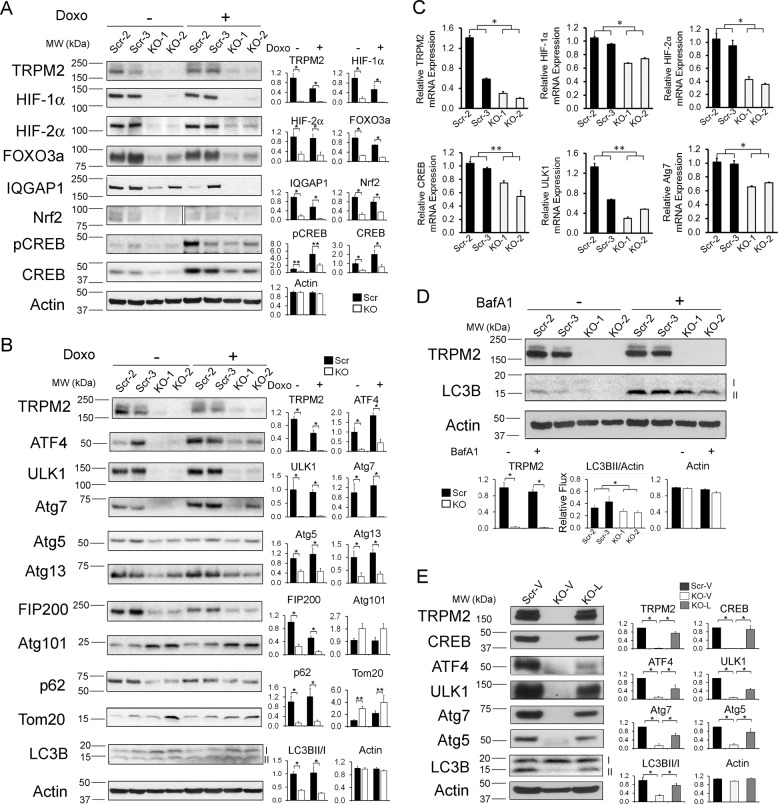
Impaired transcriptional regulation and autophagy protein expression in TRPM2-depleted U937 leukemia cells. **a** Western blotting was performed to determine expression of HIF-1α, HIF-2α, FOXO3a, IQGAP1, Nrf2, CREB, and CREB phosphorylation in TRPM2-depleted U937 cells. In these experiments, two knockout (KO-1-2) and two scrambled clones (Scr-2-3) were selected randomly for western blots with or without treatment with 0.3 µM doxorubicin. **b** Expression of autophagy proteins ULK1, Atg7, Atg5, Atg13, FIP200, Atg101, and transcription factor ATF4 was examined by western blotting. p62, Tom20, and LC3B-I and II, which are modified in autophagy, were also studied. Actin was probed to confirm equivalent loading. In **a** and **b**, western blots performed for each protein are shown on the left. One blot for each protein is shown here and the others in Supplemental Information Figs. 4, 5. Densitometry measurements from two to three experiments for each protein were standardized to results for each experiment’s average untreated scrambled control and means ± s.e.m. calculated and shown on the right (*n* = 4-6, Tom20 *n* = 8). *p ≤ 0.001, **p < 0.03, group effect, Scr vs KO, two-way ANOVA. **c** RT-PCR was used to measure TRPM2, HIF-1α, HIF-2α, CREB, ULK1, and Atg7 mRNA in TRPM2-depleted leukemia cells. Results summarizing two (TRPM2, ULK1), three (Atg7) or four (HIF-1/2α, CREB) experiments are shown (mean ± s.e.m., *n* = 8-16). *p ≤ 0.0001, **p < 0.015, one-way ANOVA. **d** U937 cell were incubated with or without bafilomycin A1, and conversion of LCB-I to II was examined with western blotting. Four experiments were performed. One blot is shown here and the others in Supplemental Information Fig. 6. Densitometry measurements were obtained. Relative autophagic flux was calculated as: (O.D. LC3B-II + Bafilomycin/O.D. Actin) - (O.D. LC3B-II-Bafilomycin/O.D. Actin) for each band. Means ± s.e.m. for the four experiments for each cell line are shown below the figure (*n* = 8). *p < 0.05, unpaired, two-tailed *t*-test. **e** Western blotting was performed to measure TRPM2, CREB, ATF4, ULK1, Atg7, Atg5, and LC3B-I and II expression in four experiments after TRPM2-L reconstitution. One blot is shown here and the others in Supplemental Information Fig. 7. Densitometry measurements were normalized to each blots’ untreated scrambled control, and mean densitometry measurements ± s.e.m. for experiments with each protein are shown on the right. *p < 0.04 (*n* = 4) one-way ANOVA.

To further examine mechanisms of reduced cell survival and mitochondrial function in TRPM2-depleted leukemia cells, expression of transcription factors involved in regulation of mitochondrial proteins and antioxidants was examined with Western blotting. Expression of HIF-1α and HIF-2α, which are regulated by calcium dependent pathways and involved in modulation of mitochondrial proteins including members of the ETC, were decreased in TRPM2-depleted U937 leukemia cells, as was the downstream transcription factor FOXO3a (Fig. 6a). Nrf2, a transcription factor which regulates expression of many cellular antioxidant genes, was also significantly decreased in TRPM2-depleted cells (Fig. 6a). IQGAP1, which stabilizes Nrf2 through a calcium dependent process, was decreased in TRPM2 depletion (Fig. 6a) and may contribute to the decrease in Nrf2 on a posttranslational level. Through mitochondrial and ETC dysfunction, the decrease in HIF-1/2α may contribute to increased ROS production and the decrease in FOXO3a and Nrf2 to reduced antioxidant capacity. Phosphorylated and total CREB, which regulate expression of proteins involved in mitochondrial function, and activating transcription factor 4 (ATF4), which has an important role in autophagy, were significantly reduced in TRPM2-depleted cells (Fig. 6a, b). To determine if these key transcription factors are reduced on a transcriptional basis, HIF-1α, HIF-2α, and CREB mRNA were quantitated. RT-PCR determined that HIF-1α, HIF-2α, and CREB are at least partially reduced on a transcriptional basis (Fig. 6c).

### Autophagy is significantly impaired by TRPM2 depletion

Autophagy is an important pro-survival mechanism which eliminates damaged mitochondria and controls levels of ROS. Because mitochondria are dysfunctional in TRPM2-depleted leukemia cells and ROS levels are increased, the effect of TRPM2 depletion on autophagy was examined. Low levels of ULK1, Atg7, and Atg5 were found in the KO (Fig. 6b). Conversion of LC3B-I to II was reduced and levels of Tom20 were increased in KO cells, confirming impaired autophagy. Another autophagy substrate, p62, may increase if autophagy is impaired; its decrease in TRPM2 KO cells may be secondary to downregulation of Nrf2, a known transcription factor regulating p62 expression.

ULK1, Atg7, and Atg5 are transcriptional targets of CREB and ATF4, suggesting autophagy may be reduced on a transcriptional basis. RT-PCR confirmed a significant decrease in CREB, ULK1, and Atg7 mRNAs, as well as HIF-1/2α mRNAs, in TRPM2-depleted cells (Fig. 6c). Atg13, FIP200, and Atg101, which play a role in ULK1 protein stability, were examined. Atg13 and FIP200 but not Atg101 were significantly lower in TRPM2-depleted cells (Fig. 6b), suggesting that the decrease in ULK1 may be secondary to both transcriptional and posttranslational mechanisms. To confirm reduced autophagic flux in TRPM2-depleted cells, the lysosomal turnover of LC3B-II was examined in the presence or absence of bafilomycin A1, a lysosomal inhibitor widely used to block autophagic degradation. While treatment with bafilomycin A1 increased LC3B-II levels in both control and KO cells, autophagic flux, as measured by the difference of LC3B-II levels between the bafilomycin A1 treated and untreated cells, was significantly reduced in TRPM2 KO cells (Fig. 6d).

Reconstitution of TRPM2 but not empty vector restored expression of CREB, ATF4, autophagy proteins ULK1, Atg7, and Atg5, and conversion of LC3B-I to II (Fig. 6e). These experiments demonstrate the key role of TRPM2 in regulation of autophagy, through both transcriptional and post translational pathways.

### AML-193 leukemia cells also demonstrated decreased proliferation and viability and reduced autophagy protein expression in TRPM2 knockdown

**Fig. 7:**
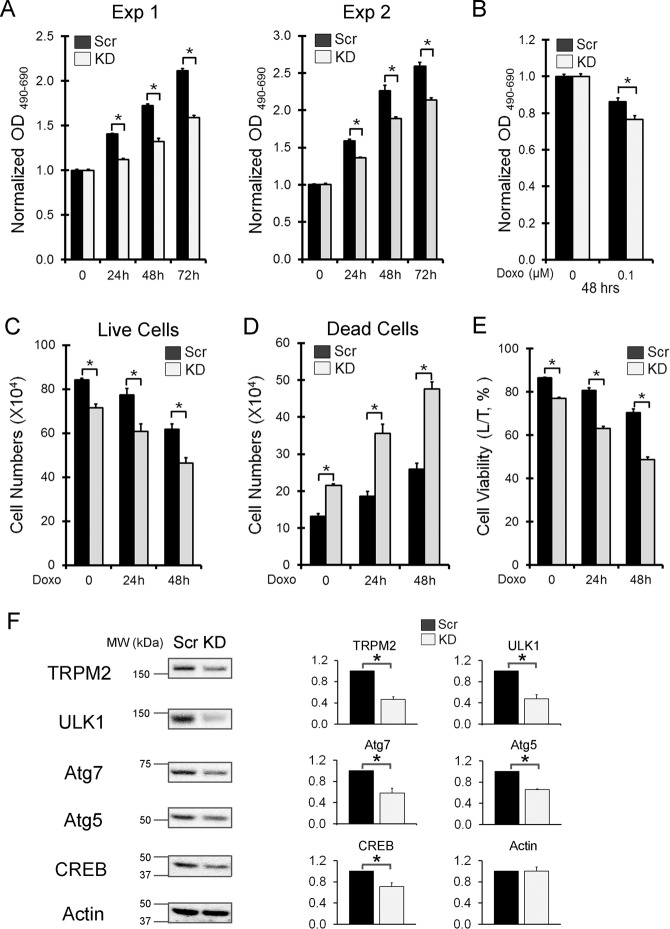
Knockdown of TRPM2 in AML-193 leukemia cells. TRPM2 was reduced in AML-193 with shRNA targeted to TRPM2 and stable transfectants were generated. Pooled knockdown and control cells were studied. **a** The equivalent numbers of AML-193 scrambled control (Scr) and TRPM2 knockdown (KD) cells were plated at time 0 in two experiments. Cell proliferation was measured with XTT at 0-72 h after plating and both experiments are shown as mean ± s.e.m. OD_490–690_ normalized for each group to time 0 cells (*n* = 4–6/experiment). *p < 0.0001, group effect, Scr vs KD; *p < 0.0001, time effect; *p < 0.0125, group × time interaction effect, two-way ANOVA, indicating that differences between groups are amplified by time. **b** Cell viability was measured at 48 h after doxorubicin (0.1 µM) exposure with XTT. Mean ± s.e.m. of OD_490–690_ normalized to time 0 from three experiments are shown for Scr and TRPM2 knockdown cells (*n*=12). *p* < 0.01, group effect, two-way ANOVA. **c**–**e** Viability was also measured after 24–48 h of treatment with 0.3 µM doxorubicin with trypan blue exclusion. Mean ± s.e.m. number of live (**c**) or dead cells (**d**) from two experiments is shown, (*n* = 16). Percent viability from the two experiments (mean ± s.e.m.) is presented in **e** (*n* = 16). In **c**–**e** *p < 0.0001, group effect, two-way ANOVA. **f** Western blots of expression of TRPM2, ULK1, Atg7, Atg5, CREB and actin with untreated scrambled or knockdown cells are shown. Densitometry measurements were normalized to each blots’ scrambled control in three experiments, and mean normalized densitometry measurements ± s.e.m. for the three are shown on the right. *p < 0.02, nonparametric, unpaired, two-tailed *t*-test. Full western blots for each antibody in all experiments are shown in Supplemental Information Fig. 8.

To confirm findings in another AML cell line, AML-193 cells were stably transfected with shRNAs targeted to TRPM2 or scrambled control shRNAs. Reduction of TRPM2 was confirmed by western blotting (Fig. 7f). Cell proliferation (Fig. 7a, two experiments) and viability at 48 h after doxorubicin (Fig. 7b) measured by XTT were significantly lower in cells in which TRPM2 expression was reduced. The number of live cells at 24 and 48 h after doxorubicin, measured by trypan blue exclusion, was significantly reduced in TRPM2-depleted cells (Fig. 7c) and the number of dead cells was significantly increased (Fig. 7d). Percent cell viability was significantly reduced in TRPM2-depleted cells (Fig. 7e). Decreased expression of ULK1, Atg7, Atg5, and CREB was also demonstrated when TRPM2 was reduced in these cells (Fig. 7f).

### Reconstitution of ATF4 or CREB in TRPM2-depleted cells restores proliferation, survival and autophagy

**Fig. 8:**
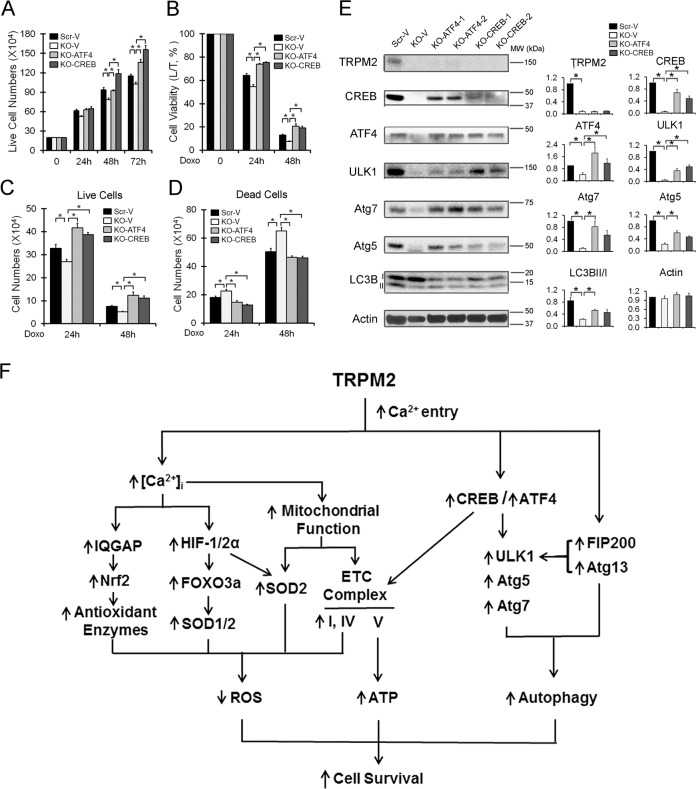
Reconstitution of ATF4 or CREB in TRPM2 in depleted cells restored cell proliferation, viability, and expression of autophagy proteins. TRPM2-depleted U937 cells were stably transfected with empty vector (KO-V), ATF4 (KO-ATF4) or CREB (KO-CREB) and 20 × 10^4^ cells were plated. **a** Proliferation was quantitated as live cell number by trypan blue exclusion over 0–72 h. Viability at 24 and 48 h after treatment with 0.3 µM doxorubicin was quantitated as **b** % viable cells, **c** live cell and **d** dead cell number. Mean ± s.e.m. is shown (*n* = 8). *p < 0.0167, subgroup analysis, Bonferroni correction, two-way ANOVA. Three additional experiments analyzed with XTT to assess viability are shown in Supplemental Information Fig. 9. **e** Western blotting was done on two or three experiments to assess TRPM2, CREB, ATF4, ULK1, Atg7, Atg5, and four for LC3B-I and II expression using two clones reconstituted with ATF4 or CREB. Densitometry measurements were normalized to each blots’ untreated scrambled control, and mean densitometry measurements ± s.e.m. for the experiments are shown on the right. One blot for each protein is shown here, and the others in Supplemental Information Fig. 9e. *p < 0.05, one-way ANOVA. **f** Schema of TRPM2 modulation of mitochondrial function, ROS production, autophagy, and cell survival in leukemia.

To determine their role in modulation of autophagy by TRPM2, TRPM2-depleted U937 cells were transfected with ATF4 or CREB. The ability of ATF4 or CREB to restore proliferation and viability of TRPM2-depleted cells treated with doxorubicin to that of scrambled control cells was examined. Data were analyzed by two-way ANOVA. Comparing all four groups in proliferation studies (Scr-V, KO-V, KO-ATF4, KO-CREB), there were significant differences in group (*p* < 0.0001), time (*p* < 0.0001), and group × time interaction effects (*p* < 0.0001), indicating that difference in proliferation between groups is amplified with time (Fig. 8a). Applying the Bonferroni correction to subgroup analyses (Scr-V vs KO-V, KO-V vs KO-ATF4, and KO-V vs KO-CREB) so that *p* < 0.0167 is statistically significant, group (*p* < 0.0001), time (*p* < 0.0001) and group × time interaction effects (*p* < 0.0122) were significantly different for all three subgroup comparisons. When cells were treated with doxorubicin, there were significant group and time effects in cell viability (Fig. 8 b), live cell number (Fig. 8c), and dead cell number (Fig. 8d). In three subgroup analyses, comparing Scr-V vs KO-V, KO-V vs KO-ATF4, and KO-V vs KO-CREB, significant differences in group (*p* < 0.0004) and time (*p* < 0.0001) (Fig. 8b, c) were achieved. The number of dead cells after doxorubicin was significantly different in all three subgroup comparisons for group (*p* < 0.0001), time (*p* < 0.0001), and group × time interaction effects (*p* < 0.009, Fig. 8d). These data demonstrate the role of ATF4 and CREB in preserving proliferation and cell viability in TRPM2 expressing cells. Expression of ULK1, Atg7, and Atg5 were examined with western blotting. ATF4 significantly restored autophagy proteins ULK1, Atg7, Atg5, and conversion of LC3B-I to II to levels above those in TRPM2-depleted cells, but only Atg7 to levels measured in Scr control (Fig. 8e). ULK1 was also significantly restored by CREB; Atg7 and Atg5 tended to be partially restored, but results did not reach statistical significance (Fig. 8e). Reconstitution of CREB also restored ATF4 expression and ATF4 restored CREB.

## Discussion

TRPM2 is highly expressed in human AML cells. When TRPM2 is depleted, AML cell proliferation and survival after anthracycline chemotherapy are reduced. Mitochondrial function and cellular bioenergetics are significantly impaired and ROS are increased due to electron transport chain (ETC) dysfunction and decreased Nrf2. In addition, TRPM2 depletion dramatically reduced expression of key autophagy associated proteins including ULK1, Atg7, and Atg5 through mechanisms involving CREB and ATF4. This work demonstrates the significant roles of TRPM2 in cell viability, mitochondrial function, ROS regulation, and autophagy in AML.

The first major finding of this report is that TRPM2 expression is increased in leukemia cells of patients with AML, suggesting TRPM2 is pro-survival. Indeed, TRPM2 depletion significantly inhibited human leukemia proliferation and increased leukemia sensitivity to doxorubicin (Fig. 3). This is consistent with reports in neuroblastoma^31^, T-cell ALL^40^, and gastric cancer^38^ in which TRPM2 depletion reduced malignant cell survival. It differs from a report examining AML generated on a Trpm2-/- genetic background in murine hematopoietic cells induced by retroviral introduction of MLL-AF9 or BCR-ABL-GFP and NUP98-HOXA9-YFP^41^. Loss of TRPM2 in that model had no major effect on leukemogenesis or potentiation of cytotoxic therapy. That models differs from the one reported here in that the leukemia was induced in murine cells with retroviral vectors encoding aggressive genetic subtypes, and those mice had time to develop compensatory pathways.

The second major finding here is that mitochondrial function is significantly decreased and ROS increased in TRPM2-depleted human myeloid leukemia cells, particularly after doxorubicin exposure. A proposed schema of pathways through which TRPM2 sustains mitochondrial function and cell survival is shown on Fig. 8f. Mitochondrial proteins including those involved in ETC complexes, oxygen consumption, ATP production, and mitochondrial calcium uptake are significantly reduced in TRPM2 depletion and contribute to decreased leukemia survival and increased susceptibility to doxorubicin^10,21,31^. Decrease in transcription factors including HIF-1/2α, FOXO3a, and CREB in TRPM2-depleted leukemia cells, a third finding of this report, contributes to ETC dysfunction through reduced ETC protein expression, increasing mitochondrial ROS and reducing ATP. In addition, a steady, low level of Ca^2+^ uptake by mitochondria is essential for maintenance of cellular bioenergetics and is decreased with TRPM2 depletion^42^.

Nrf2 is a transcription factor which regulates expression of over 200 genes including many antioxidants^43^. Reduction in Nrf2 and accumulation of ROS restricts self-renewal of hematopoietic stem cells^44^. While Nrf2 protects non-malignant cells from oxidative stress and transformation by activation of antioxidant enzymes, it also protects malignant cells from oxidative stress and cytotoxic chemotherapy^45^. A major finding of this report is that Nrf2 is significantly reduced in TRPM2 depletion in leukemia, contributing to increased ROS and chemotherapy sensitivity^43^. Calcium impacts Nrf2 signaling through IQGAP1, a GTPase activating protein which stabilizes Nrf2^46^. Increased intracellular calcium enhances translocation of Nrf2 and the Nrf2/IQGAP1 complex into the nucleus, increasing Nrf2 mediated target gene transcription. In contrast, silencing of IQGAP1 decreases Nrf2 expression and expression of Nrf2 target genes. In TRPM2-depleted leukemia cells, expression of IQGAP1 is decreased, contributing to reduced Nrf2 stability. These data show that the ROS increase in TRPM2-depleted cells results both from increased production by compromised ETC and an impaired antioxidant response. Of note, increased intracellular ROS may also contribute to mitochondrial dysfunction through oxidation of mitochondrial proteins^47^.

The fourth major finding is that through modulation of transcription factors CREB and ATF4, TRPM2 regulates expression of ULK1, Atg7 and Atg5, significantly impacting autophagy. ROS induce autophagy, which eliminates damaged mitochondria and controls cellular ROS levels^48^. Autophagy is a pro-survival mechanism in hematopoietic disease and promotes resistance to chemotherapy^32^ whereas blockade of autophagy increases chemotherapy sensitivity in AML^32,36^. Autophagy and ATF4 play a key role in FLT3-ITD mutation mediated leukemia proliferation and survival^49^. Autophagy inhibition or downregulation of ATF4 overcame FLT3 inhibitor resistance and inhibited cell proliferation. In TRPM2 depletion, autophagy is suppressed, contributing to accumulation of dysfunctional mitochondria, increased ROS and anthracycline sensitivity, and cell death. These data suggest that TRPM2 inhibition may also have a therapeutic role in AML through inhibition of autophagy.

Autophagy is regulated by the ULK1 complex, which plays a key role in autophagosome formation^50,51^ and ULK1 mRNA levels positively correlate with reduced relapse-free survival and therapeutic resistance in breast cancer and nasopharyngeal carcinoma^51,52^. In TRPM2-depleted cells, ULKI is reduced by transcriptional and post translational mechanisms^53^. ULK1 transcription may be affected by both decreased ATF4 and CREB. ULK1 is a target of ATF4, a master regulator of transcription of genes involved in adaptation to stress, which is suppressed in TRPM2 depletion^51,53^. Nrf2 is a positive regulator of ATF4, and reduced Nrf2 may contribute to decreased ATF4^54^. Atg5 may also be modulated indirectly through DNA damage inducible transcript 3 (DDIT3), which is regulated by ATF4^53^. The ULK1 promoter has several putative CREB sites, and CREB is downregulated in TRPM2-depleted leukemia cells. CREB also regulates other autophagy genes including Atg7 and Atg5, which are decreased in TRPM2-depleted cells^53,55^. Reconstitution experiments show ATF4 and CREB contribute to reduced ULK1, Atg7, and Atg5 in TRPM2 depletion and can partially restore autophagy protein expression. Of note, reconstitution of CREB expression restored ATF4, and expression of ATF4 restored CREB. While both CREB and ATF4 proteins could bind each others putative CRE binding sites to enhance expression^56^, indirect activation through other transcription factors, binding to other proteins including CREB binding protein, or posttranslational mechanisms may contribute to the reciprocal relationship. In addition, the FOXO family of transcription factors participates in ULK1 regulation and FOXO3a is downregulated in KO cells^53^. The mechanisms through which TRPM2 regulates these transcription factors are under investigation. TRPM2 also modulates ULK1 through post translational mechanisms^51,57,58^. ULK1 is part of a multimeric protein complex composed of Atg13, RB1/FIP200, and Atg101, which are phosphorylated by ULK1^57^ and are important for ULK1 stability and kinase activity. Atg13 and FIP200 are reduced in TRPM2-depleted leukemia cells^50,58^, and this may contribute to reduced ULK1 stability.

The work presented here demonstrates the important role of TRPM2 in regulation of mitochondrial function, ROS production, bioenergetics, and autophagy in proliferation and survival of myeloid leukemia. TRPM2 contributes to modulation of these processes through regulation of transcription factors including HIF-1/2α, Nrf2, ATF4, and CREB, and the autophagy regulators ULK1, Atg7, and Atg5. Together, these data strongly suggest that TRPM2 modulates a number of vital processes synchronously in AML, and targeted inhibition of TRPM2 is an important and novel therapeutic approach.

## Materials and methods

### TRPM2 expression in AML patient samples, leukemia cell lines, and hematopoietic precursors

AML patient samples were obtained from the Institute of Personalized Medicine (IPM) at the Pennsylvania State University College of Medicine under protocols approved by the Institutional Review Board. Consent was obtained at sample collection by the IPM tumor bank and was not required for this study by the Institutional Review Board because samples were de-identified. Human myeloid leukemia cell lines and neuroblastoma cell line SH-SY5Y were purchased from American Type Culture Collection (ATCC, Manassas, VA, USA). Human bone marrow CD33 + and CD34+ cells were purchased from STEMCELL Technologies (Vancouver, BC, Canada). The Bloodspot BloodPool Data Set, AML samples with normal cells, was utilized to examine TRPM2 expression in HSCs (Lin^−^/CD34^+^/CD38^−^/CD90^+^/CD45RA^−^), CMPs from normal human bone marrow (CMP), and AML samples with different cytogenetic mutations^59^. Western blotting was performed as described.

### Depletion of TRPM2

U937 cells were depleted of endogenous TRPM2 with CRISPR (Clustered regularly-interspaced short palindromic repeats) technology as described^31^. Multiple single cell clones were generated and expanded. TRPM2 depletion was confirmed with RT-PCR and western blotting. Three clones with TRPM2 depletion were chosen at random for experiments. In reconstitution experiments, U937 cells in which TRPM2 was depleted were transfected with wild type TRPM2 subcloned into pcDNA3.1/V5-His TOPO plasmid, ATF4 or CREB subcloned into pCMV6-Entry vector, or empty vector using the Neon Transfection System (Invitrogen, USA). Stably transfectants were generated and maintained in 200 µg/ml G418 (Gemini Bio-Products, West Sacramento, CA, USA). Plates were treated with doxorubicin (0.1 to 0.5 µg/ml) when cells were 70–80% confluent. For xenograft experiments, TRPM2-depleted and scrambled U937 cell lines were generated which express luciferase and tdTomato using the pCDH-EF1-Luc2-P2A-tdTomato lentiviral plasmid (Addgene, Cambridge, MA, USA). TRPM2 currents were measured in TRPM2-depleted or scrambled control cells with whole cell patch-clamp as described^31,60^. Cell proliferation and viability were assessed using XTT (2,3-Bis(2-methoxy-4-nitro-5-sulfophenyl)-2H-tetrazolium-5-carboxanilide) proliferation assay (Trevigen Inc., Gaithersburg, MD, USA) or by cell counting with trypan blue exclusion.

In some experiments, TRPM2 was reduced in AML-193 cells by infection with lentivirus expressing shRNA targeted to TRPM2 (Origene, Rockville, MD, USA). Cells infected with lentivirus were enhanced by selection with cell sorting for GFP, expressed by the lentivirus vector, and are a pooled population. U937 and AML-193 cells were utilized here because they are frequently used in experiments on myeloid leukemia, they showed detectable levels of TRPM2 by Western blotting, and knockout clones and/or knockdown cell lines were successfully generated.

### RT-PCR of endogenous TRPM2, ULK1, Atg7, and CREB expression in leukemia

TRPM2 mRNA was quantitated in leukemia samples and cell lines, and normal CD33+/CD34+ precursors as described^21^. Primers/probes for HIF-1α (Assay ID Hs00153153-m1), HIF-2α (Assay ID Hs01026149-m1), ULK1 (Assay ID Hs00177504-m1), Atg7 (Assay ID Hs00893766-m1), CREB (Assay ID Hs00231713) and TATA Binding Protein Control (ID Hs00427620) were from Applied Biosystems (Waltham, MA, USA).

### TRPM2-depleted leukemia xenografts

For myeloid leukemia xenografts, 8–12 weeks old female NSG (NOD.Cg-Prkdc^scid^Il2rg^tm1Wjl^/SzJ) mice (Jackson Laboratory, Bar Harbor, ME, USA) were injected intravenously via tail vein with 8 ×10^3^ luciferase and tdTomato positive U937 cells depleted of TRPM2, or U937 scrambled control cells. Twelve mice per group were used in each of five experiments. After injection of luciferin, images of luciferin intensity in mice were measured twice weekly using a IVIS Lumina III CT Living Image 4.5 machine (Perkin Elmer), and relative luminescence units were calculated. Tissues were harvested at experiment completion. Sample size was chosen based on previous experiments^31^. No blinding to group allocation or randomization of mice was done. All protocols were approved by and in compliance with policies of the Institutional Animal Care and Use Committee of Pennsylvania State University College of Medicine.

### Immunoblot analysis

Whole cell lysates were prepared and subjected to 4–20% sodium dodecyl sulfate-polyacrylamide gradient gel electrophoresis (SDS-PAGE)^21^. Blots were probed with anti-TRPM2-C (1:300; Bethyl Laboratories, Montgomery, TX, USA); with antibodies to ATF4 (1:400), Atg7 (1:2000), Atg13 (1:500), Atg101 (1:500), pCREB1 and CREB1 (1:250), FIP200 (1:300), forkhead box transcription factor 3a (FOXO3a; 1:400), HIF-1α (1:250), Nrf2 (1:400), and ULK1 (1:1000) from Cell Signaling Technology (Boston, MA, USA); Atg5 (1:1000) from Medical Biological Lab (Japan); COX6B1 (1:500), MT-CO2 (1:500), MT-ND2 (1:500), and NDUFA13 (1:300) from LSBio (Seattle, WA, USA); HIF-2α (1:500) and LC3B (1:2000) from Novus (Littleton, CO, USA); IQGAP1 (1:5000) from Abcam (Cambridge, MA, USA); p62 (1:5000) from American Research Products (Waltham, MA, USA); Tom20 (1:5000) from Santa Cruz Biotech (Dallas, TX, USA); and actin (1:5000) from Sigma (St. Louis, MO, USA). Blots were incubated with horseradish peroxidase (HRP)-conjugated antibodies (1:2000). Enhanced chemiluminescence was used for detection of signal and intensity of bands quantitated with densitometry. In all cases, gels were transferred to filter paper, and the filters were cut into small molecular weight spans prior to probing. The entire portion of the western blot which was probed which each antibody is shown, except for TRPM2, which had a significant amount of empty space above the TRPM2 band. Figures were prepared with Adobe Photoshop Elements 7.

### Measurements of mitochondrial function

Mitochondrial membrane potential (Δψ_m_) was measured with JC-10 Mitochondrial Membrane Potential Assay Kit or the TMRE Kit (Abcam), using flow cytometry and manufacturer’s instructions. Mitochondrial Ca^2+^ uptake, oxygen consumption rate (OCR), ATP levels, and mitochondrial ROS were measured as described^21,31^.

### Proteomic analysis

For label-free proteomics studies, extracted proteins were digested with trypsin^21^. Mass spec analysis was performed using QE mass spectrometer as described previously^61^.

### Statistical analysis

Results are expressed as mean ± s.e.m.. The sample size for each experimental group and a statement of the number of experiments performed in each figure subsection is provided in figure legends. For analysis of TRPM2 current as a function of group and voltage, two-way analysis of variance (ANOVA) was used. For analysis of protein expression levels, O_2_ consumption, XTT, trypan blue exclusion, ATP levels, and ROS levels, two-way ANOVA, one-way ANOVA or *t*-tests were used as indicated. In analyses, *p* ≤ 0.05 was considered statistically significant or as specified when Bonferroni correction applied to two-way ANOVA analysis.

## Supplementary information

Supplemental Information Figure Legends

Supplemental Figure 1

Supplemental Figure 2

Supplemental Figure 3

Supplemental Figure 4

Supplemental Figure 5

Supplemental Figure 6

Supplemental Figure 7

Supplemental Figure 8

Supplemental; Figure 9

## Data Availability

The data generated during and/or analyzed in the current study are available from the corresponding author on reasonable request. Supplementary Information is available at the Cell Death and Disease’s website.
