# Supplementary material for: Transient receptor potential ion channel TRPM2 promotes AML proliferation and survival through modulation of mitochondrial function, ROS, and autophagy
Source: Cell Death Dis. 2020 Apr 20;11(4):247. doi: 10.1038/s41419-020-2454-8 (PMC7170900; doi:10.1038/s41419-020-2454-8)
Supplement: Supplementary file 1 — Supplemental Information Figure Legends [file 41419_2020_2454_MOESM1_ESM.docx]

**Supplemental Information Figure Legends**

**Supplemental Figure 1**

**A, C-I (Related to Figure 3 A, C-I)** (A, C-E) Additional experiment with U937 scrambled control (Scr-1-3) and TRPM2 KO clones (KO-1-3) for (A) proliferation (trypan blue exclusion), (C) viability after doxorubicin (live cell number, trypan blue), (D) dead cell number, and (E) % viable cells. *p<0.0001, group effect (Scr vs KO), two-way ANOVA. (F-I) Additional experiment showing effects of TRPM2 reconstitution with vector (V) or TRPM2 (TRPM2-L) on (F) proliferation and (G-I) viability after treatment with 0.3 µM doxorubicin, analyzed with trypan blue exclusion. (F, H) live cell number; (G) % viable cells; (I) dead cells. *p<0.008, subgroup analysis, Bonferroni correction, two-way ANOVA.

**Supplemental Figure 2**

**(Related to Figure 3 J, K)** Two xenograft experiments of NSG mice injected with control or TRPM2 KO U937 cells in addition to the one in Figure 3 J-K are shown. Mice were injected with luciferin and luminescence quantitated with the IVIS System. K shows mean + s.e.m. luminescence. Second experiment terminated at day 14 for tissue harvest. *p analyzed with unpaired, two-tailed t-test.

**Supplemental Figure 3**

**(Related to Figure 5 H)** Full blot images used to create Figure 5H are shown. Sections used for untreated or doxorubicin treated cells were spliced in order to show different exposure times (COX6B1, NDUFA13, MT-ND2) or to remove wild-type lanes (MT-CO2). Sections used are indicated with black boxes.

**Supplemental Figure 4**

**(Related to Figure 6 A)** Additional Western blots of scrambled control (Scr-2,3) and TRPM2 depleted (KO-1,2) U937 cells probed with antibodies to TRPM2, HIF-1α, HIF-2α, FOXO3, IQGAP1, Nrf2, phosphorylated CREB, CREB, or Actin and used for densitometry in Figure 6A. Boxes indicate area of blot used. Cells were untreated (-) or treated with 0.3 µM doxorubicin (+). Nrf2 blots had lanes in which wild type (Wt) parental U937 cells were run. For the Nrf2 blot on the right, used in Figure 6A, Wt lane was removed in the preparation of the figure. Boxes indicate where the lane was spliced together after removal of lanes representing wild type cells.

**Supplemental Figure 5**

**(Related to Figure 6 B)** Additional Western blot experiments of scrambled control (Scr) and TRPM2 depleted (KO) U937 cells probed with antibodies to TRPM2, ATF4, ULK1, Atg7, Atg5, Atg13, FIP200, Atg101, p62, Tom20, LC3B, and Actin, and used for densitometry in Figure 6B. Boxes indicate area of blot used. Cells were untreated (-) or treated with 0.3 µM doxorubicin (+).

**Supplemental Figure 6**

**(Related to Figure 6 D)** Scrambled control (Scr-2,3) and TRPM2 depleted (KO-1,2) U937 cell were incubated with or without bafilomycin A1. Conversion of LCB-I to II was examined. Western blots were probed with antibodies to TRPM2, LC3B, and Actin. All three experiments used are shown here. Boxes indicate area of blot used for densitometry in Figure 6D.

**Supplemental Figure 7**

**(Related to Figure 6 E)** Effect of TRPM2 reconstitution with vector (V) or TRPM2 (TRPM2-L) on autophagy protein expression. Western blotting was performed with antibodies to TRPM2, CREB, ATF4, ULK1, Atg7, Atg5, and LC3B in three additional experiments after TRPM2-L reconstitution. Densitometry measurements were obtained, normalized, and included in densitometry analysis in Figure 6E.

**Supplemental Figure 8**

**(Related to Figure 7 F)** Effect of TRPM2 knockdown in AML-193 cells on autophagy protein expression. After stable transfectants of TRPM2 knockdown with shRNA were generated, Western blotting was performed with antibodies to TRPM2, ULK1, Atg7, Atg5, CREB, and Actin. All experiments are shown. Boxes indicate areas of blots used for densitometry in Figure 7F.

**Supplemental Figure 9**

**XTT (Related to Figure 8)** Effect of ATF4 and CREB reconstitution on cell viability. KO, reconstituted, and control U937 cells were treated with 3 µM doxorubicin for 24 or 48 hours. Mean + s.e.m. OD_490-690_ measured with XTT and normalized to untreated control cells in three experiments is shown. *p<0.0167, subgroup analysis, Bonferroni correction, two-way ANOVA.

**E (Related to Figure 8 E)** Effect of reconstitution of TRPM2 KO cells with vector (V), ATF4 (KO-ATF4) or CREB (KO-CREB) on autophagy protein expression. Western blotting was performed with antibodies to TRPM2, CREB, ATF4, ULK1, Atg7, Atg5, and LC3B in additional experiments. Densitometry measurements were normalized and included in densitometry analysis in Figure 8E.
