# Supplementary figures and images for: Transient receptor potential ion channel TRPM2 promotes AML proliferation and survival through modulation of mitochondrial function, ROS, and autophagy
Source: Cell Death Dis. 2020 Apr 20;11(4):247. doi: 10.1038/s41419-020-2454-8 (PMC7170900; doi:10.1038/s41419-020-2454-8)

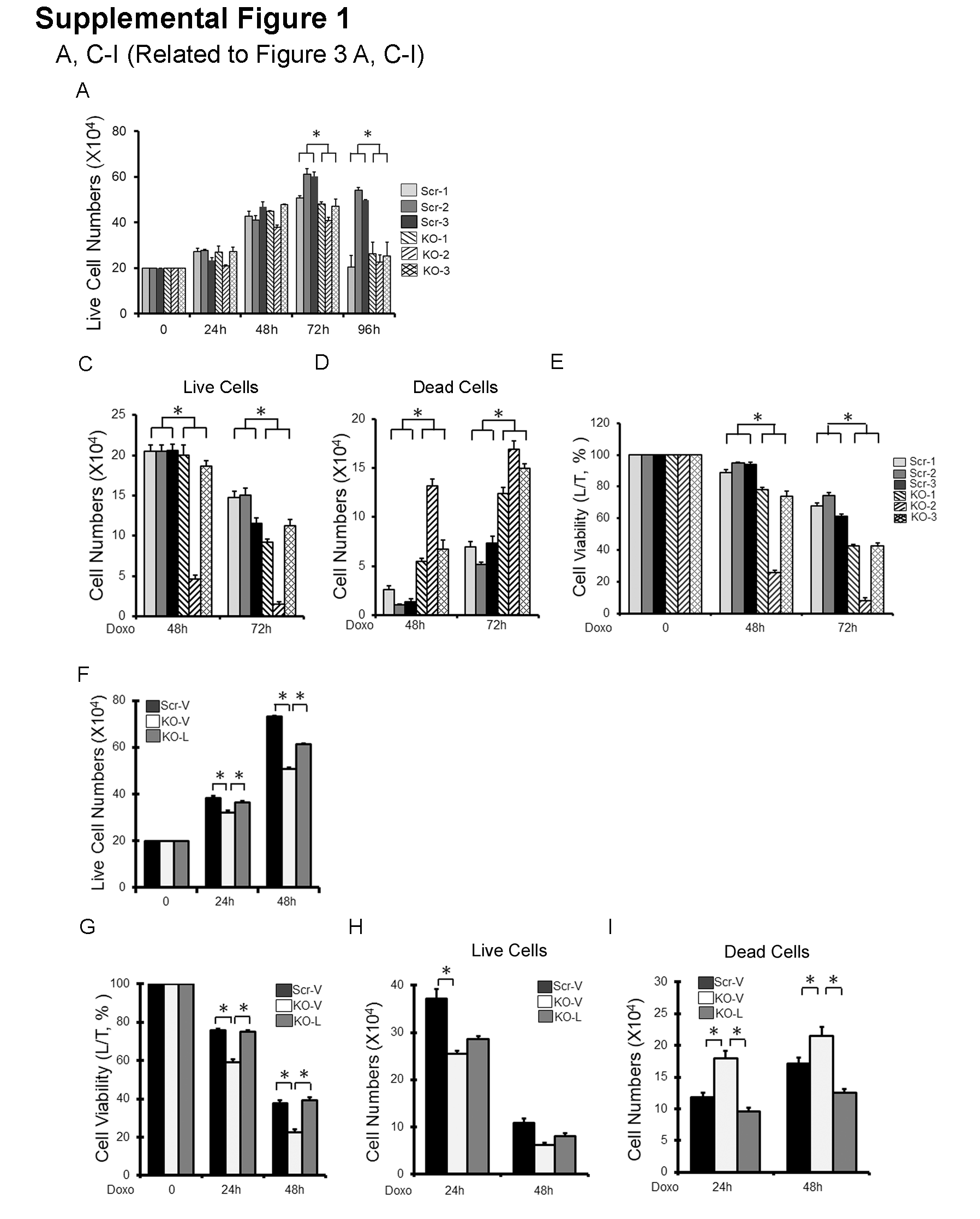

Supplement: Supplementary file 2 — Supplemental Figure 1 [file 41419_2020_2454_MOESM2_ESM.tif]

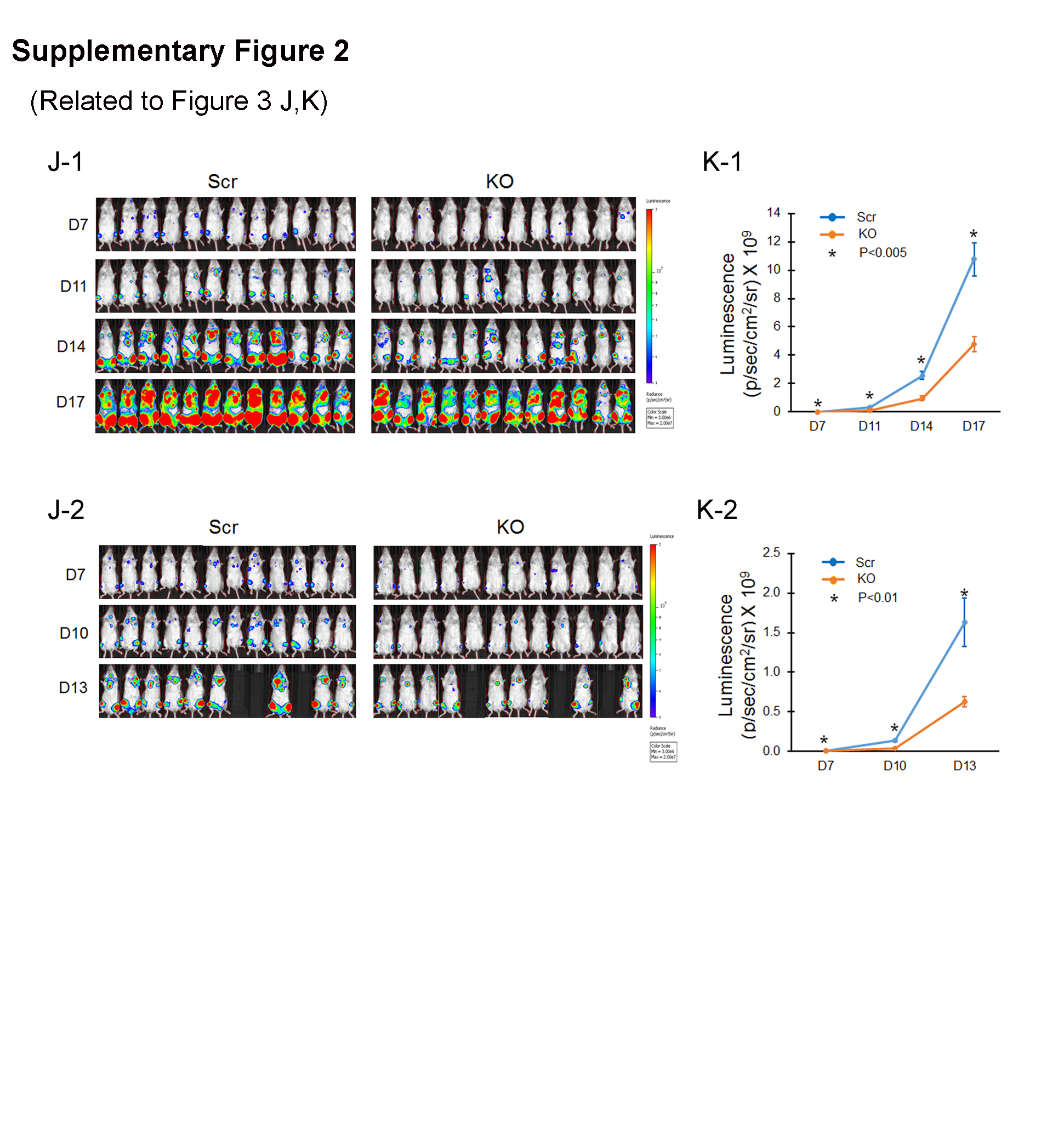

Supplement: Supplementary file 3 — Supplemental Figure 2 [file 41419_2020_2454_MOESM3_ESM.tif]

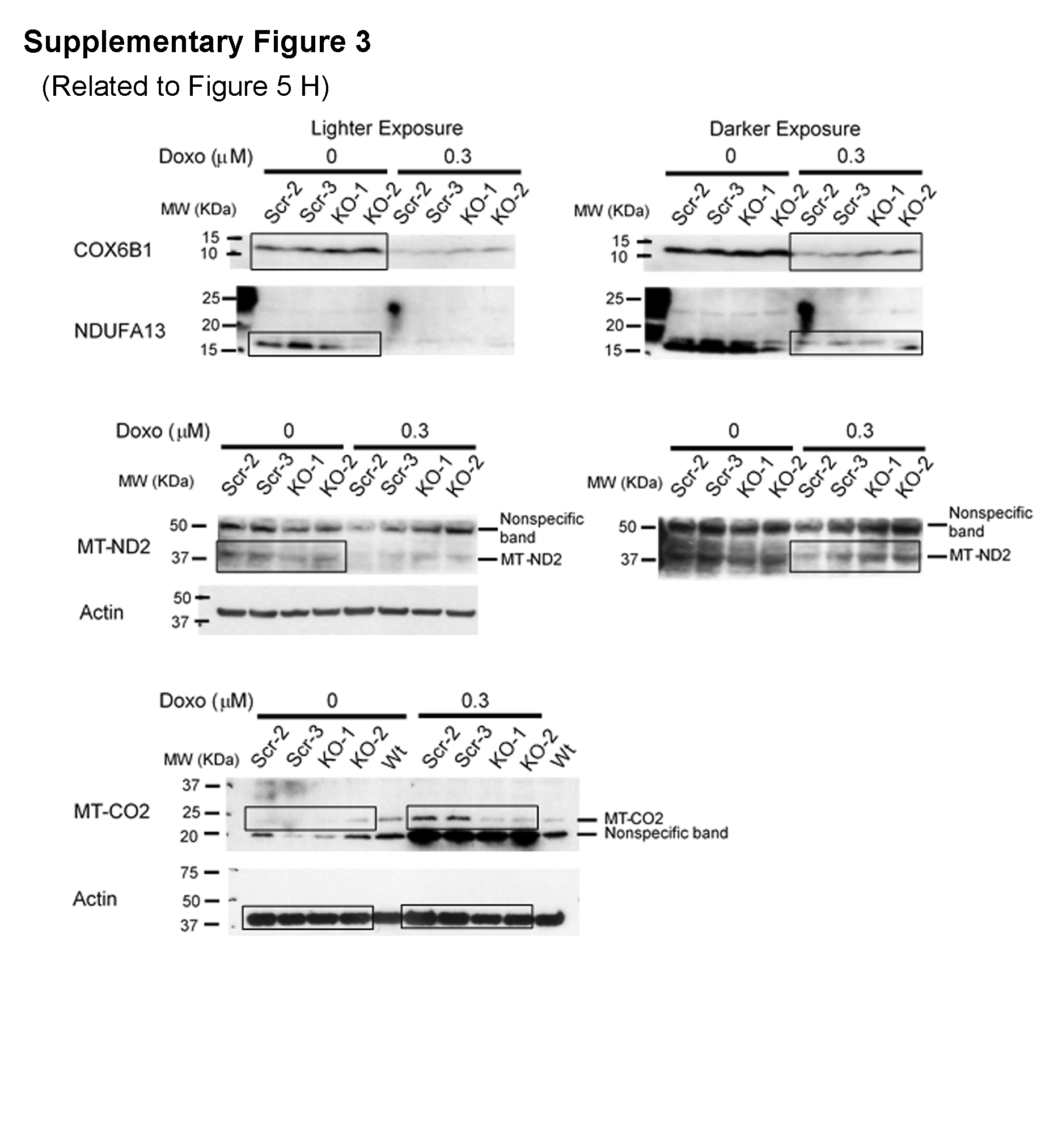

Supplement: Supplementary file 4 — Supplemental Figure 3 [file 41419_2020_2454_MOESM4_ESM.tif]

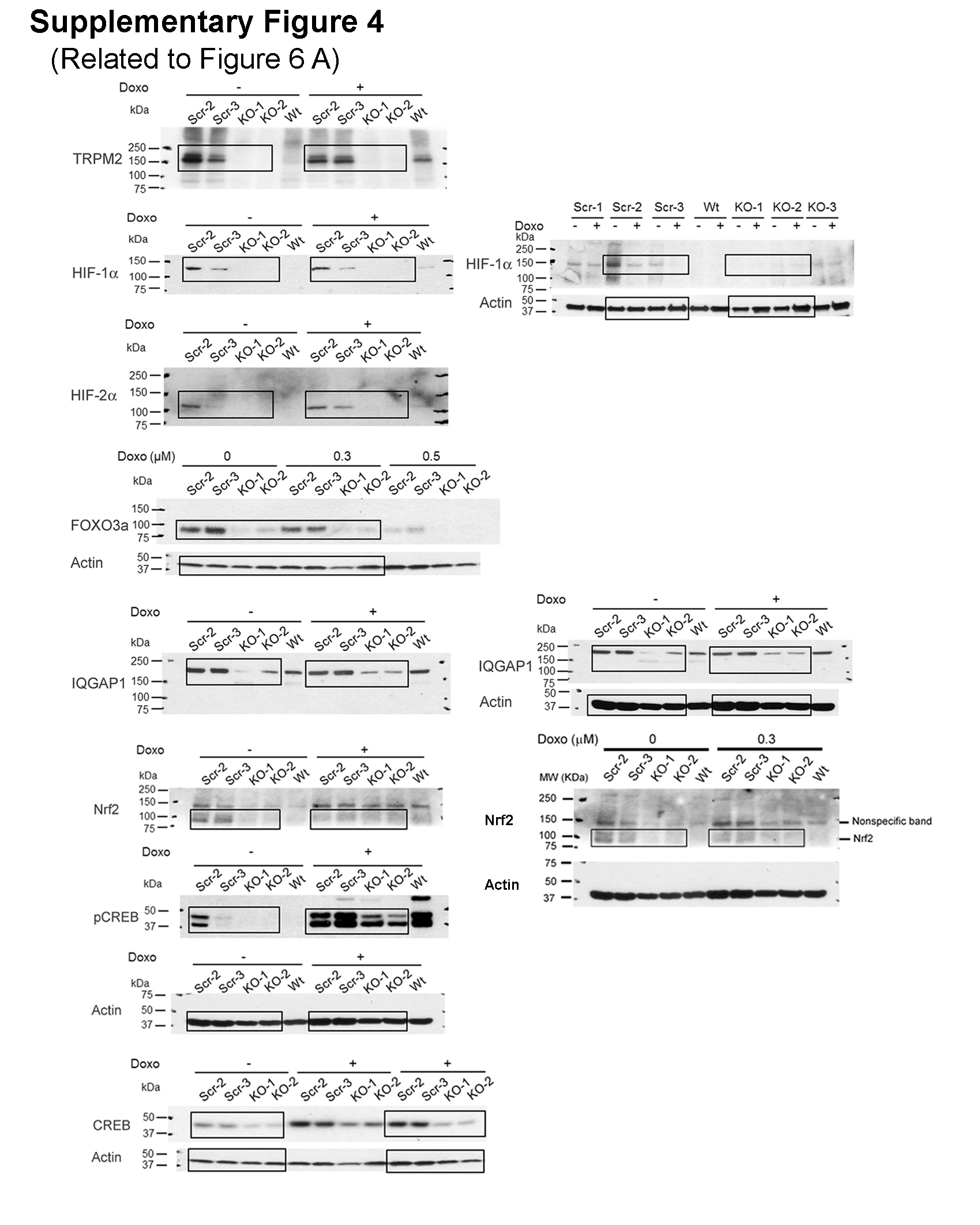

Supplement: Supplementary file 5 — Supplemental Figure 4 [file 41419_2020_2454_MOESM5_ESM.tif]

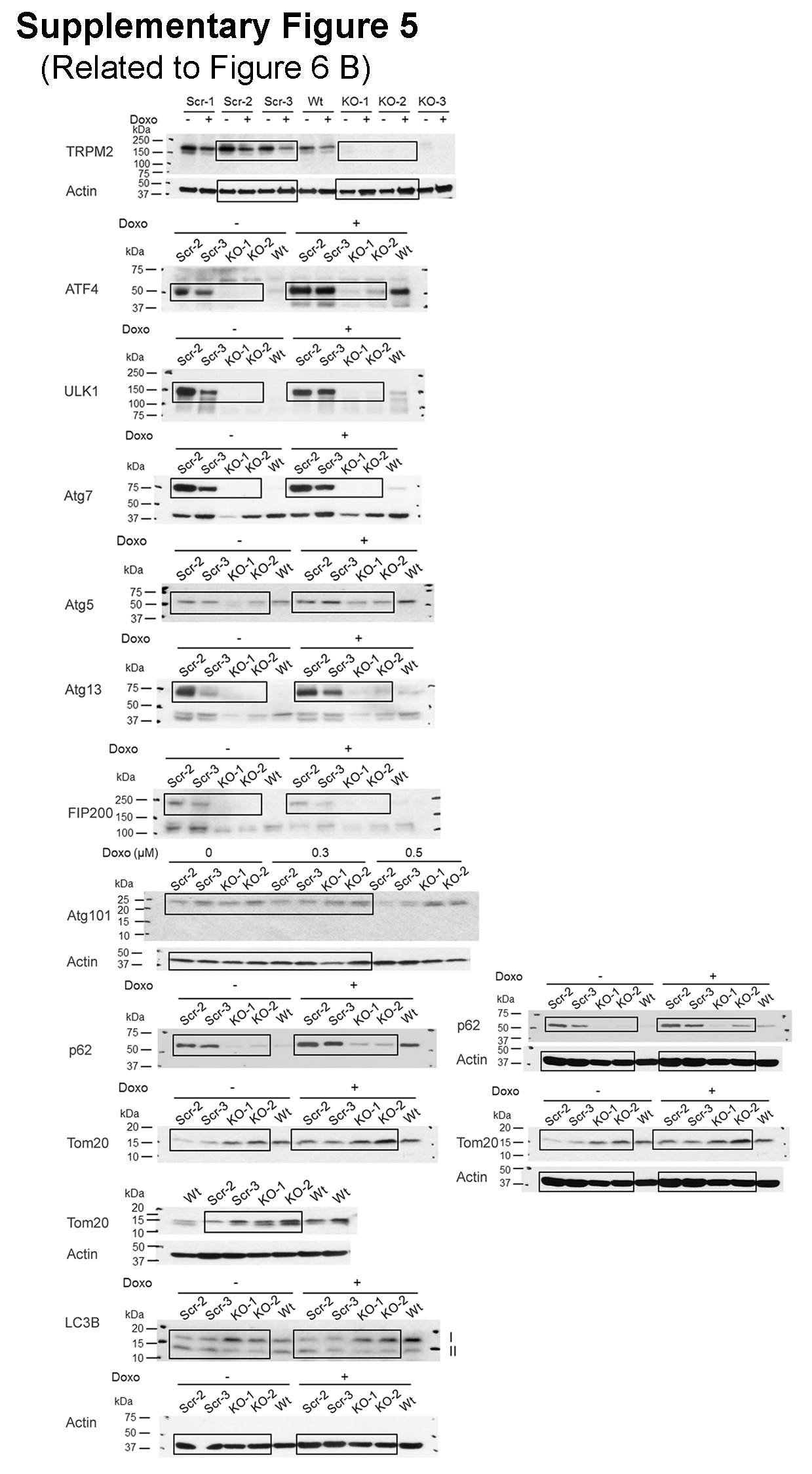

Supplement: Supplementary file 6 — Supplemental Figure 5 [file 41419_2020_2454_MOESM6_ESM.tif]

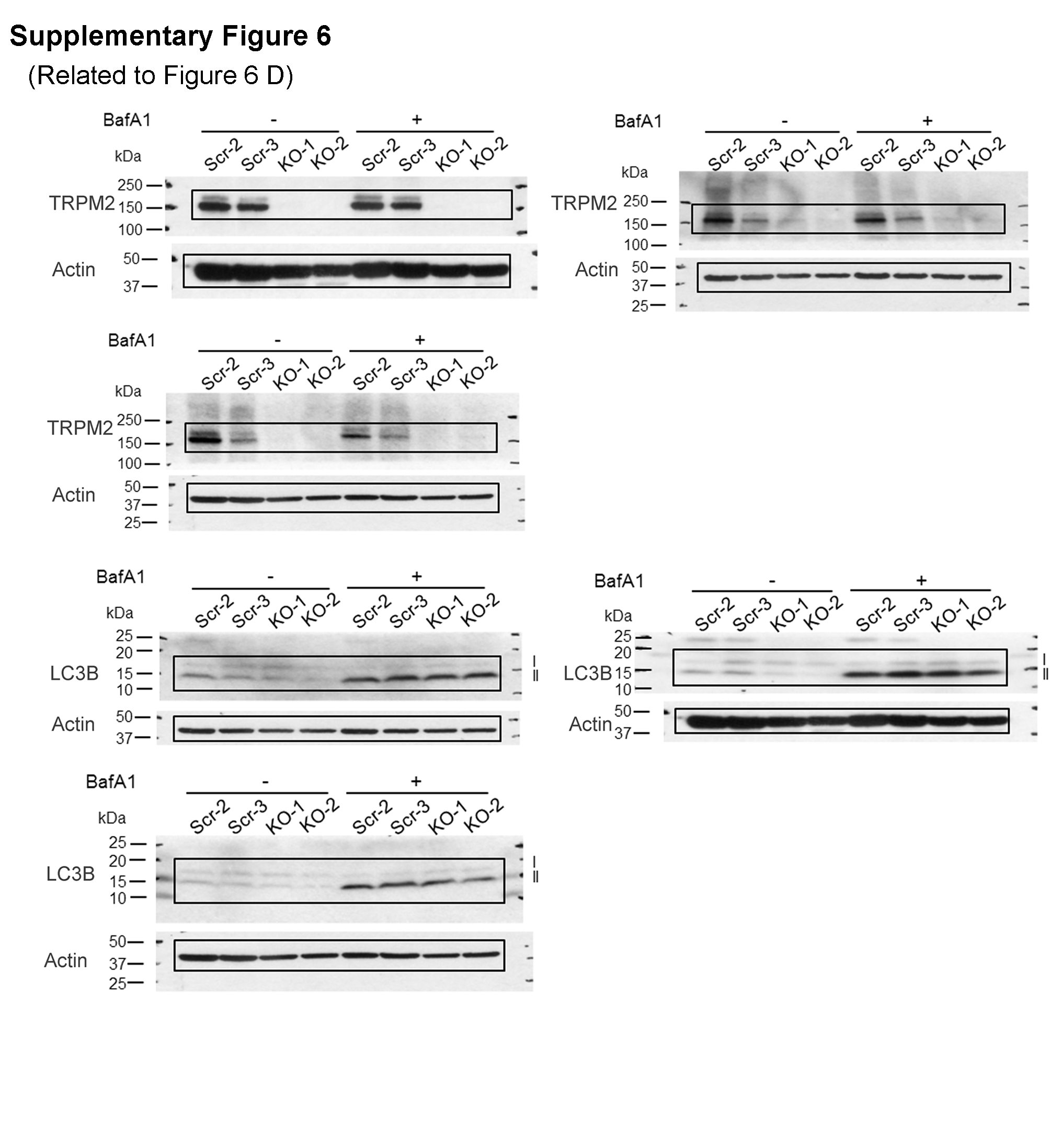

Supplement: Supplementary file 7 — Supplemental Figure 6 [file 41419_2020_2454_MOESM7_ESM.tif]

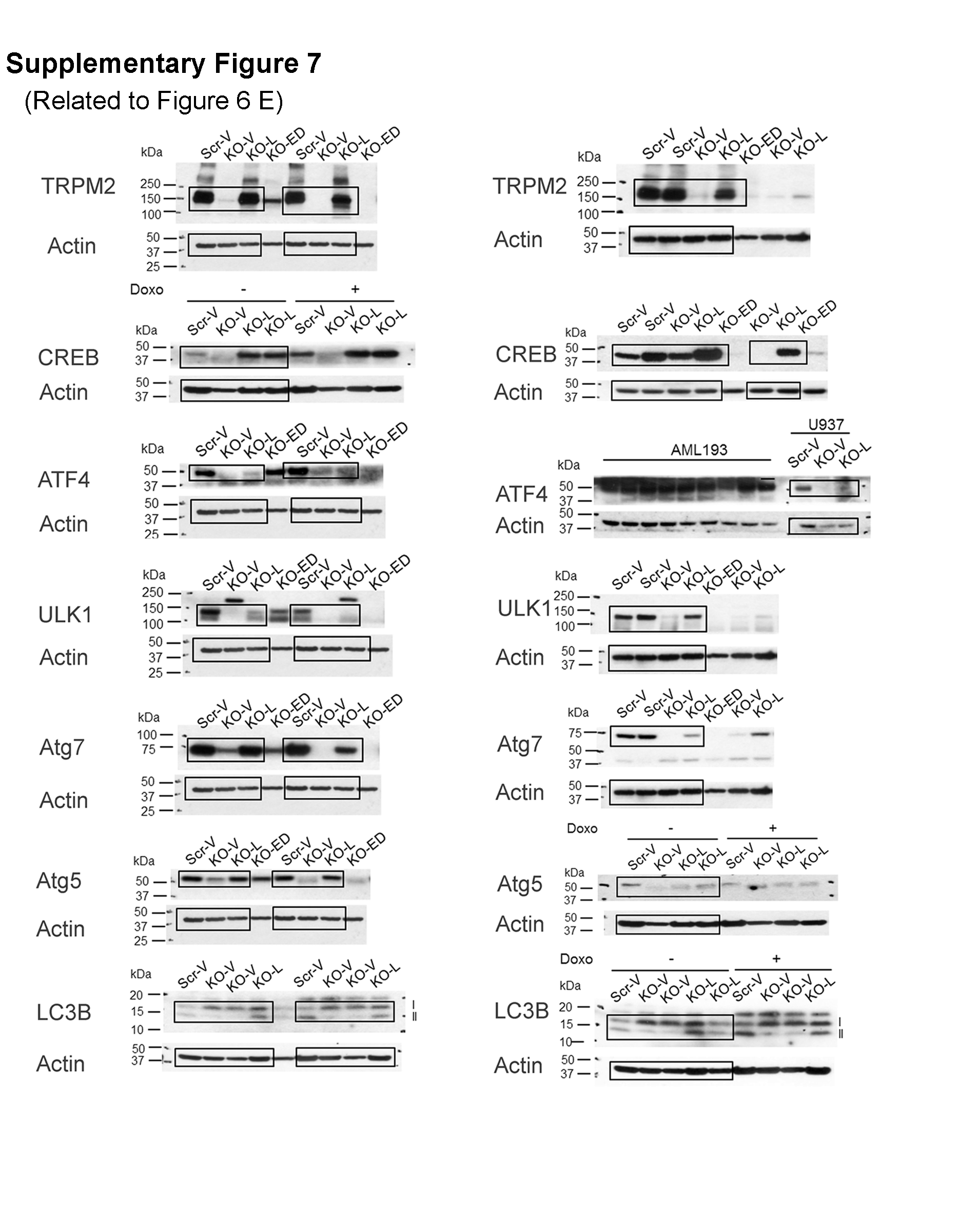

Supplement: Supplementary file 8 — Supplemental Figure 7 [file 41419_2020_2454_MOESM8_ESM.tif]

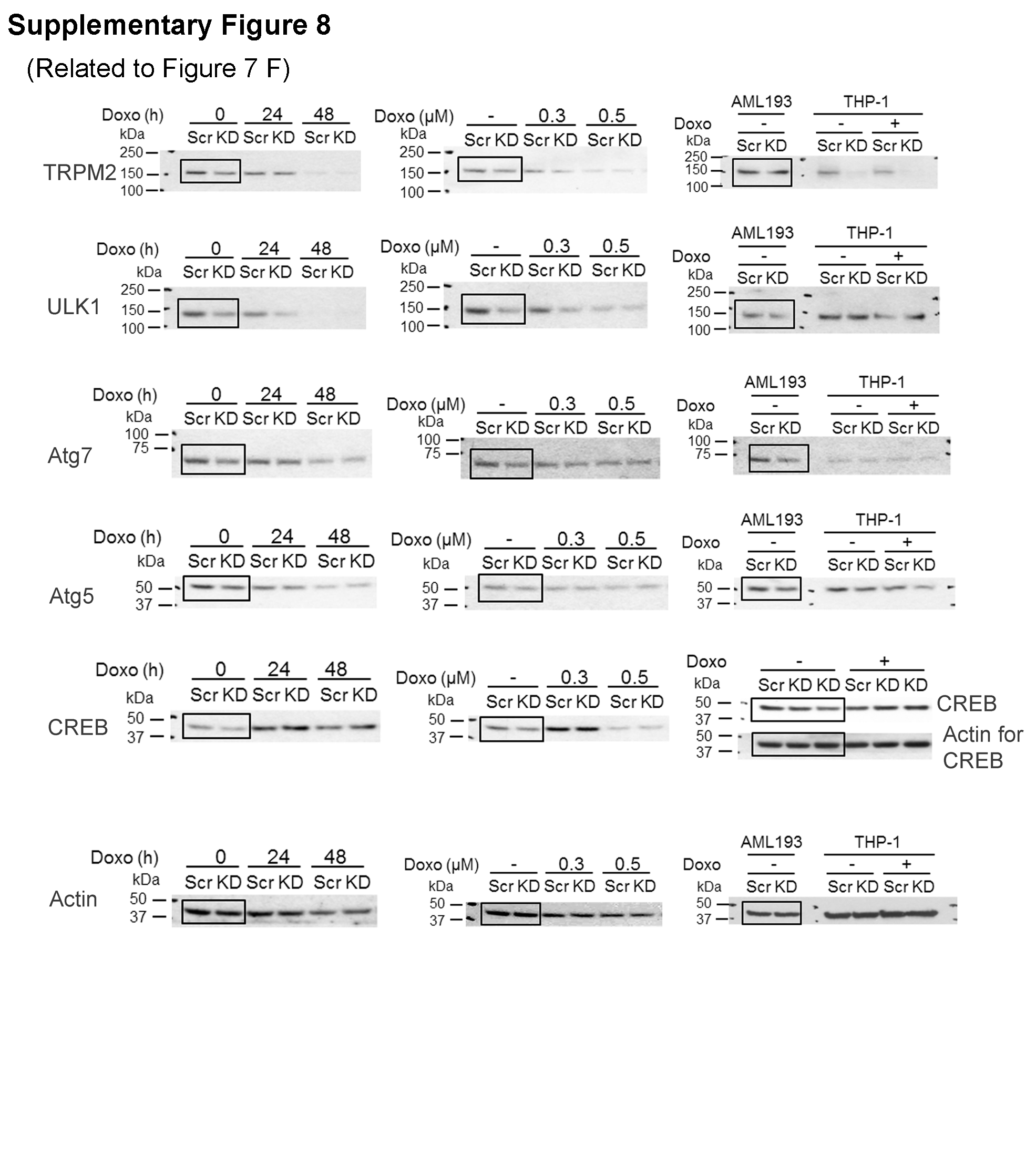

Supplement: Supplementary file 9 — Supplemental Figure 8 [file 41419_2020_2454_MOESM9_ESM.tif]

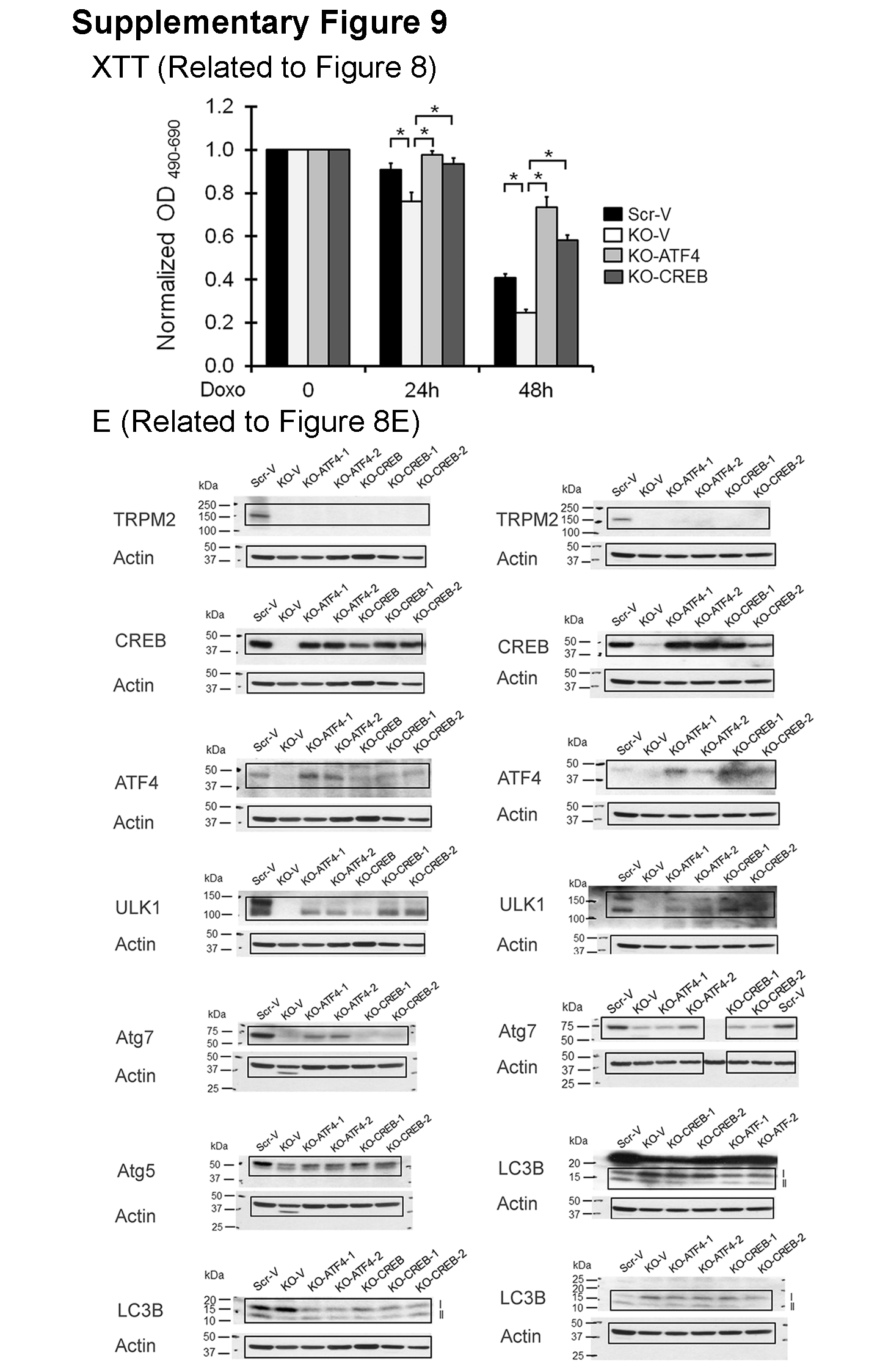

Supplement: Supplementary file 10 — Supplemental; Figure 9 [file 41419_2020_2454_MOESM10_ESM.tif]
